# MicroRNA-145-5p inhibits the tumorigenesis of breast cancer through SENP2-regulated ubiquitination of ERK2

**DOI:** 10.1007/s00018-024-05505-8

**Published:** 2024-11-23

**Authors:** Xu Chen, Danqing Li, Qi Su, Xing Ling, Siyu Ding, Runxiao Xu, Zhaoxia Liu, Yuanyuan Qin, Jinping Zhang, Zhihui Yang, Xunlei Kang, Yitao Qi, Hongmei Wu

**Affiliations:** 1 College of Life Sciences, Shaanxi Normal University, Xi’an, Shaanxi China; 2 Department of Pathology, The Affiliated Hospital of Southwest Medical University, Luzhou, Sichuan China; 3 Center for Precision Medicine, Department of Medicine, University of Missouri School of Medicine, Columbia, MO USA

**Keywords:** SENP2, ERK2, MicroRNA-145-5p, SUMOylation, Breast cancer

## Abstract

Breast carcinoma exhibits the highest incidence among various cancers and is the foremost cause of mortality in women. Increasing evidence shows that SUMOylation of proteins plays a critical role in the progression of breast cancer; however, the role of SENP2 and its molecular mechanism in breast cancer remain underexplored. Here, we discerned that SENP2 promoted the tumorigenesis of breast cancer both in vitro and in vivo. Furthermore, we identified that ERK2 was SUMOylated and that SENP2 played a role by deconjugating ERK2 SUMOylation in breast cancer. SUMOylation of ERK2 promoted its ubiquitin-proteasomal degradation, thus inhibiting the epithelial-to-mesenchymal transition in breast cancer cells. Furthermore, microRNA-145-5p (miR-145-5p) has emerged as a scarce commodity in breast cancer and binds to the 3’-untranslated region of SENP2 mRNA to govern the regulatory dynamics of SENP2 expression. Finally, miR-145-5p inhibits SENP2 transcription, enhances ERK2 SUMOylation, and ultimately suppresses the progression of breast cancer. These revelations suggest evolving ideas for the miR-145-5p-SENP2 axis in therapeutic intervention, thus heralding transformative prospects for the clinical management of breast cancer.

**Supplementary Information:**

The online version contains supplementary material available at 10.1007/s00018-024-05505-8.

## Introduction

Breast carcinoma is the most prevalent malignancy afflicting women worldwide and comprises 15% of new cancer cases in China [1] and 30% of new female cancer cases in America [2], which poses a severe threat to the health of women. Breast cancer, marked by its heterogeneity, is rooted in histological attributes, delineating four distinct subtypes, including luminal A, luminal B, human epidermal growth factor receptor 2 (HER2)-positive, and notorious triple-negative breast cancer (TNBC) [3]. TNBC, due to its high metastasis and aggressiveness, has a survival rate of only 14% among advanced patients [4]. Although current therapies for breast cancer are diversified, the cure rate is still low due to drug resistance. Therefore, the exploration of new therapeutic targets has been the focus of breast cancer research.

Intriguingly, we found that small ubiquitin-like modifier (SUMO) proteins constitute a distinctive class within the proteomic landscape [5]. The journey of a SUMO protein precursor is cleaved and then followed by maturation under the catalytic guidance of SUMO-specific protease (SENP), activated by the activating enzyme, transitioning to the conjugating enzyme before culminating in its ligation to the designated target protein, under the meticulous orchestration of the ligating enzyme [6]. SUMOylation, characterized by its multifaceted modulatory potential, functions as an essential regulator governing an array of physiological and pathological processes [7]. A constellation of six SENPs, each endowed with distinct substrate specificities, collectively govern this intricate landscape, encompassing SENP1-3 and SENP5-7 [8]. SENP2 assumes a multifaceted role in cardiac development [9], myogenesis [10], neurogenesis [11], sudden unexplained death in epilepsy [12, 13], and spinal muscular atrophy-like pathology [14]. SENP2 has cast its shadow across a spectrum of malignancies, spanning bladder cancer [15] and hepatocellular carcinoma [16]. Although prior investigations have shed light on SENP2 participation in the realm of breast cancer cells, illuminating its regulatory role in glucose glycolysis [17]. Although the molecular mechanisms by which SENP2 is involved in breast cancer is beginning to be understood [18], further elucidation is necessary.

ERK2, also known as MAPK1, regulates various cellular processes by phosphorylating multiple target proteins [19]. ERK2 was reported to play crucial roles in the development of breast cancer in vivo [20]. ERK2 activation triggers cell differentiation and stimulates cell survival and is an attractive target for pharmacotherapy [21]. RAF and MEK inhibitors are used for clinical treatment; however, no ERK inhibitor is being developed [22]. Hence, delving into the intricacies of ERK2 inhibition in breast cancer emerges as a venture of significance.

Recent studies have highlighted the pivotal role of non-coding RNA in the pathogenesis of cancer, and their emerging importance as biomarkers and therapeutic targets in breast cancer. Notably, recent studies have uncovered a diverse transcriptional profile of PRMT1-derived circRNAs [23] and elucidated the complex regulatory roles of non-coding RNA in the cell cycle progression of breast cancer [24], underpinning our investigation into specific non-coding RNAs and their potential as novel therapeutic targets of breast cancer. MicroRNA-145-5p (miR-145-5p) is a critical gene regulator with substantial implications in breast tumorigenesis [25]. Previous investigations have suggested that miR-145-5p plays a role in suppressing breast cancer invasion by partially silencing the metastasis-associated gene mucin 1 [26]. Another study showed that the circIQCH-miR-145-DNMT3A axis plays a critical role in breast cancer cell growth and metastasis through competing endogenous RNAs [27]. Nonetheless, the role of miR-145-5p in the regulation of post-translational modifications governing breast cancer progression remains obscure.

In the current study, we elucidated that SENP2 meticulously affects the migration and invasion of breast cancer cells. Examination of putative SENP2 target proteins revealed that ERK2 is primarily SUMOylated by SUMO2 and that SENP2 deconjugates the SUMOylation of ERK2, increasing the stability and activity of ERK2 and ultimately promoting the epithelial-to-mesenchymal transition (EMT) and the progression of breast carcinoma. Furthermore, we determined that miR-145-5p suppressed SENP2 transcription and then inhibited cell growth and metastasis of breast cancer. In total, our studies reveal the crucial role played by SENP2 in ERK2 deSUMOylation and discover an essential role of the SENP2-ERK2 axis in the progression of breast cancer, with profound implications for the expansive domain of clinical breast cancer therapeutics.

## Materials and methods

### Cell culture and treatment

Human embryonic kidney cells HEK293T (RRID: CVCL_0063), human luminal A breast cancer cells MCF-7 (RRID: CVCL_0031) and T47D (RRID: CVCL_0553), human TNBC cells BT-549 (RRID: CVCL_1092), MDA-MB-231 (RRID: CVCL_0062) and MDA-MB-468 (RRID: CVCL_0419), and mouse breast cancer cells 4T1 (RRID: CVCL_0125) were purchased from Servicebio company. These cells were meticulously nurtured in a conducive environment, specifically in Dulbecco’s modified Eagle medium (DMEM, Thermo, MA, USA), fortified with 10% fetal bovine serum (FBS, Thermo, MA, USA), 100 µg/ml streptomycin, and 100 units/ml penicillin, all within the confines of a controlled incubation chamber maintained at a temperature of 37 °C, and replete with a humidified atmosphere infused with 5% CO_2_. The siRNA oligonucleotides targeting SENP2, miR-145-5p mimic and inhibitor were purchased from GenePharma (Supplementary Table S1). Cells were transiently transfected with Lipofectamine 2000 (Invitrogen) according to the manufacturer’s protocol. Briefly, the DNA and Lipofectamine 2000 was diluted in Opti-MEM medium (Thermo, MA, USA) and incubated for 5 min. The DNA and Lipofectamine 2000 were mixed and allowed to form complex for 20 min at room temperature and then added to the cells. The transfected cells were cultured in a humidified incubator for indicated experiments. All human cell lines have been authenticated using STR profiling within the last three years, and all experiments were performed with mycoplasma-free cells.

### Xenograft mouse models

Five-week-old female BALB/c SCID mice were purchased from Beijing Vital River Laboratory Animal Technology and used for experiments. All mice were housed at room temperature (22 °C), with a 12-hour light-dark cycle and ad libitum access to food and water. sh-SENP2 or control MDA-MB-231 cells (4 × 10^6^) were subcutaneously implanted into the left flank of SCID mice. The growth of tumors was determined by measuring of the dimensions of the xenograft every 4 days before reaching a mean volume of 100 mm^3^. The body weight of the mice was recorded every 4 days, and the mice were monitored each day. Tumor volumes were calculated with the formula: volume (mm^3^) = 0.5 × longest tumor diameter × (shortest tumor diameter)^2^. At the endpoint, the mice were euthanized, and the tumors were collected for weight and Western blotting analysis. The animals were housed in 2 to 5 mice per cage. All animal studies were performed according to the protocol “Guide for the Care and Use of Laboratory Animals”, which was approved by the esteemed Institutional Animal Care and Use Committee of Shaanxi Normal University, and all manipulations were carried out according to established guidelines.

### Breast cancer specimens and immunohistochemistry (IHC) staining

Breast cancer patients were diagnosed at the Affiliated Hospital of Southwest Medical University, and the detailed clinical data were listed in Supplemental Table S7. For our investigations, tissue microarray chips comprising a total of 48 pairs of tumors and their corresponding adjacent tissues were procured from Biotech Well (Supplemental Table S8). It is essential to underscore that these tumor and adjacent non-cancerous tissue specimens were originally collected during surgical procedures and were obtained primarily for diagnostic and therapeutic purposes. Paraffin-embedded sections of 10 randomly selected breast cancer specimens were performed by IHC staining of SENP2 and ERK2 expression by an IHC kit (Vector Laboratories, CA, USA) according to the manufacturer. The staining results were scored by multiplying the percentage classification by the intensity [28]. All human specimens were analyzed for the current study with appropriate IRB approved by the Affiliated Hospital of Southwest Medical University, and the studies abide by the Declaration of Helsinki principles. The GEPIA2 database (http://gepia.cancer-pku.cn) was applied to perform differential expression analysis of target genes between tumor and normal samples, as well as survival analysis to investigate the prognostic value of gene expression levels in breast cancer patients. The last date of access to the platform was October 30, 2023 with email address at qiyitao@snnu.edu.cn.

### Construction of plasmid

The eukaryotic expression plasmids harboring wild-type RGS-His-SENP2, the catalytic mutant RGS-His-SENP2, HA-SUMO1, and HA-SUMO2 have been comprehensively elucidated in prior literature [9, 12]. The FLAG-ERK2 plasmid was kindly provided by Dr. Qiao Wu from Xiamen University, and the SUMO site mutation of ERK2 was generated using the Quick-change site-directed mutagenesis kit (Vazyme, Nanjing, China) with the indicated primers (Supplementary Table S2). All plasmids were confirmed by DNA sequencing (Supplementary Table S3).

### Generation of lentivirus and transduction

The sh1-SENP2 and sh2-SENP2 knockdown cell lines were generated using the lentiviral system (System Biosciences, CA, USA). Lentiviral expression plasmids, including pCDH-SENP2, wild-type and SUMOylation site mutant pCDH-ERK2, were generated via a standard PCR-based strategy with the indicated primers (Supplementary Table S2). sh-NC was purchased from System Biosciences, and sh-SENP2 plasmids were kindly provided by Dr. Jinke Cheng in Shanghai Jiao Tong University. The lentivirus was generated by HEK293TN cells by co-transfecting the plasmids along with package (psPAX2) and envelope (pMD2.G) plasmids. Cell culture medium was harvested 2 days after transfection and transduced into target cells. The successfully transduced cells were selected by puromycin incubation for 2 days. Real-time PCR and immunoblotting (IB) were performed to confirm the efficiency of gene knockdown or overexpression.

### Dual-luciferase reporter assay

The potential interacting sites of miR-145-5p with the 3′-untranslated region (UTR) of SENP2 mRNA were constructed and cloned between the BamHI and XbaI sites of the pGL3-Promoter luciferase expression vector (Promega, WI, USA). Wild-type or mutant pGL3-SENP2 vectors were constructed. Subsequently, HEK293T cells were co-transfected with wild-type or mutant pGL3-SENP2 and then treated with miR-145-5p mimic or negative control. After 2 days of co-transfection, luciferase activity was measured using the Dual-Luciferase Reporter Assay System (Beyotime, Beijing, China). Renilla luciferase activity was used for normalization.

### Immunocytochemistry staining

Cells transfected with the specified plasmids were cultured on coverslips. Subsequently, the cells underwent a series of procedures, including washing with PBS, fixation using 4% paraformaldehyde (PFA, Thermo, MA, USA), permeabilization with Triton X-100, and incubation with the designated antibodies. Mitigation of non-specific antibody binding was achieved through treatment with 10% goat serum (Thermo, MA, USA) under ambient conditions. The primary antibody was diluted within Triton X-100 and subjected to incubation at 37 °C for 1 h. After this, the cells underwent triple PBS washes, followed by incubation for 1 h at ambient temperature with secondary antibodies labeled with Alexa Fluor 488 or 546 fluorophores (Thermo, MA, USA). The cells underwent a tripartite process involving a series of PBS washes, incubation with DAPI for 15 min, and subsequent mounting utilizing the anti-fade mounting solution (Maokangbio, Shanghai, China). Next, they were subjected to examination using confocal laser scanning microscopy (Leica, Wetzlar, Germany).

### Fluorescence in situ hybridization

Briefly, paraffin sections of breast cancer and adjacent non-cancerous tissues were deparaffinized with xylene and rehydrated with ethanol. The sections were then treated with antigen retrieval buffer for 10 min and protease K for 15 min. The sections were incubated with pre-hybridization buffer for 1 h, followed by incubation with the miR-145-5p probe at 37 °C overnight. Subsequently, the sections were washed with ice-cold PBS, incubated with DAPI for 10 min and then detected by confocal scanning microscopy (Nikon, Tokyo, Japan).

### Cell viability assay

Cellular seeding took place at a density of 2000 cells per well within 96-well plates, followed by an incubation period in a suitable culture medium. Following an incubation period of 1, 2, 3, and 4 days, cell viability of control and stably transfected cells was determined employing 3-(4,5)-dimethylthiazol-2-yl)-2,5-diphenyltetrazolium bromide (MTT, Sigma, MO, USA) in accordance with the manufacturer’s protocol. Concisely, a solution consisting of 10 µL of 4 mg/mL MTT was introduced into 100 µL of culture media. Following a 3-hour incubation at 37 °C, 100 µL of dimethyl sulfoxide (DMSO, Thermo, MA, USA) was administered to each well. Absorbance measurements were conducted employing a multimode microplate reader (Thermo, MA, USA).

### Colony formation ability assay

Cells were seeded in a 24-well plate at approximately 300 cells per well. After incubation overnight, the cells were treated with the indicated agents and incubated at 37 °C with 5% humidified CO_2_ for two weeks. The medium was changed every three days. The formed colonies were washed twice with cold PBS, fixed with methanol for 15 min, stained with crystal violet, and then detected by confocal laser scanning microscopy (Nikon, Tokyo, Japan).

### Wound healing assay

Cells were seeded in 6-well plates at 6 × 10^5^ cells per well. When the cells reached 70% confluence, the cell monolayer was gently scratched with a sterile 100 µl pipette tip to generate a mechanical wound. The cells were cultured in serum-free media at different times. The images were captured with an inverted microscope, and the scratch area was measured with ImageJ software. Average scratch width = scratch area/length. Relative width = 48-hour scratch width/0-hour scratch width.

### Transwell invasion assay

The assay for cell migration was conducted employing a 24-well Boyden chamber transwell insert (BD Biosciences, CA, USA). The Matrigel matrix (Corning, NY, USA) at a concentration of 200 µg/mL was introduced into the upper chamber. A total of 1 × 10^5^ cells, suspended in 150 µL of serum-free DMEM, were introduced into the upper chamber. The lower chamber of each well was filled with 400 µL of DMEM supplemented with 10% FBS. Following an incubation period of 24 h in a controlled environment at 37 °C with 5% CO_2_, cells that migrated to the lower surface of the upper chamber membrane were subsequently stained with 0.1% crystal violet and subsequently visualized using confocal laser scanning microscopy (Leica, Wetzlar, Germany).

### mRNA isolation and real-time PCR

To conduct a quantitative analysis of gene expression, we employed the RNeasy kit (Qiagen) to extract total mRNA from either cultured or transfected cells. Following extraction, the RNA underwent DNase treatment (Promega, WI, USA), and its concentration was quantified through absorbance measurement at 260 nm. An equivalent quantity of RNA (1 mg) served as the substrate for the synthesis of complementary DNA, a process facilitated by the high-capacity cDNA reverse transcription kit (Thermo, MA, USA). Quantitative real-time PCR was conducted employing reaction mixtures comprising cDNA, primers as specified (Supplementary Table S4), and SYBR Green reagent (Thermo, MA, USA). The ABI StepOne system (Perkin-Elmer, MA, USA) served as the platform for this analysis. The thermal protocol consisted of an initial denaturation step at 95 °C for 10 min, followed by 38 cycles of denaturation at 95 °C for 15 s, annealing and amplifying at a temperature specific to the primer sets used 60 °C for 30 s. Upon completion of the cycles, a final elongation step at 72 °C for 5 min ensured full extension of all amplified products. Real-time PCR was conducted in triplicate, and standard deviations, indicative of experimental errors, were subsequently calculated. Data analysis was executed employing ABI PRISM software (Perkin-Elmer, MA, USA). This software facilitates the determination of the threshold cycle, signifying the cycle number at which the fluorescence intensity significantly exceeds that of the background fluorescence.

### Western blotting and immunoprecipitation

The transfected cells were harvested and subsequently homogenized under chilled conditions utilizing lysis buffer containing a protease inhibitor (Targetmol, Shanghai, China). Total protein content was quantified through the utilization of the BCA assay (Servicebio, Beijing, China). Equimolar quantities of protein (20 µg in each lane) were subjected to electrophoretic separation and subsequently transferred onto PVDF membranes by electroblotting. The PVDF membrane was blocked with 5% nonfat dried milk, followed by overnight incubation with primary antibodies diluted with BSA (Supplementary Table S5). Subsequent washing was carried out, followed by incubation with peroxidase-conjugated secondary antibodies. Protein levels were subsequently determined with a chemiluminescence system (Qinxiang, Shanghai, China) after additional TBST washing steps. In the context of immunoprecipitation (IP) experiments, whole cell lysates were subjected to overnight incubation with antibodies as specified. All incubations were rigorously carried out at a temperature of 4 °C with continuous agitation. The protein complex, bound by antibodies, was effectively captured through the introduction of protein A/G agarose (refer to Supplemental Table S6), followed by an additional 2-hour incubation. After centrifugation, the protein A agarose pellet was obtained, and the IP protein complex was subsequently eluted with SDS-PAGE sample buffer, followed by Western blotting analysis utilizing the specified antibody.

### Statistical analysis

GraphPad Prism (USA) was used to evaluate the data. All data are presented as the means ± S.E.M., and experimental procedures were independently conducted in a minimum of three separate investigations. Group comparisons were conducted utilizing Student’s t test for pairwise comparisons, one-way ANOVA followed by Tukey’s or Dunnett’s test for multiple-group comparisons, or two-way ANOVA followed by Bonferroni test for comparisons involving more than two groups. Statistical comparisons were made to assess variances between groups, with statistical significance defined as *p* < 0.05 (* *p* < 0.05, ** *p* < 0.01, *** *p* < 0.001, and **** *p* < 0.0001).

## Results

### SENP2 is highly expressed and closely correlated with breast cancer patients

**Fig. 1:**
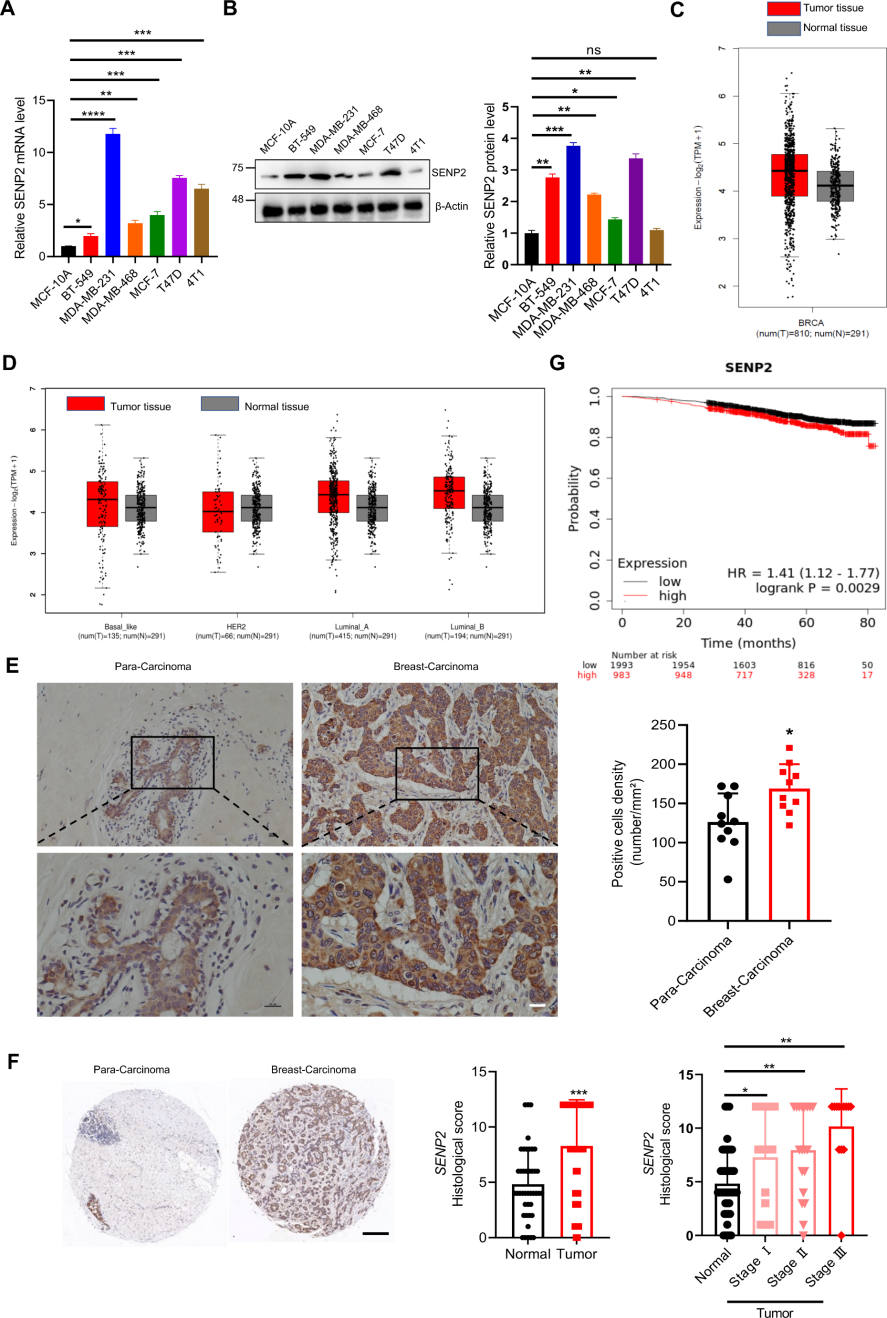
SENP2 is highly expressed in breast cancer cells and patients. **A**. The expression levels of SENP2 transcripts in normal breast cell MCF-10 A and different breast cancer cells. The quantification of SENP2 transcript levels was carried out across a range of breast cancer cell lines through real-time PCR. These values were subsequently normalized to those of BT-549 cells (*n* = 3 biological replicates per group). **B**. The expression levels of the SENP2 protein in normal breast cell MCF-10 A and different breast cancer cells. Whole cell lysates derived from multiple breast cancer cell lines underwent immunoblot analysis utilizing anti-SENP2 and anti-β-Actin antibodies (left panel). The quantitative analysis of Western blotting results is displayed in the right panel (*n* = 3 biological replicates per group) **C**. SENP2 was highly expressed in breast cancer tissue. SENP2 expression levels in breast cancer and normal tissue were analyzed with the GEPIA2 database, and the data did not show statistically significant difference. **D**. SENP2 was highly expressed in different subtypes of breast cancer tissues. SENP2 expression levels in various subtypes of breast cancer tissues were analyzed with the GEPIA2 database, and the data did not show statistically significant difference. **E**. SENP2 was highly expressed in breast cancer patients. SENP2 in breast cancer and precancerous tissue from breast cancer patients was analyzed by immunohistochemistry with SENP2 antibody (*n* = 10 biological replicates per group). Hematoxylin (blue) was applied to visualize the nuclei. The scale bar is 50 μm. **F**. SENP2 was highly correlated with the progression of breast cancer patients. SENP2 in breast cancer and precancerous tissue chips from breast cancer patients was analyzed by immunohistochemistry with the anti-SENP2 antibody (*n* = 48 biological replicates per group). The scale bar is 200 μm. **G**. SENP2 was highly correlated with overall survival in breast cancer patients. The relationship between SENP2 expression and survival rate in breast cancer patients was analyzed by survival analysis with the Kaplan-Meier Plotter database

Previous investigations have established that SENP2 assumes an anti-tumorigenic role across a spectrum of malignancies [15, 16, 29, 30]. To identify the effect of SENP2 on the tumorigenesis of breast cancer, our inquiry extended to the analysis of SENP2 expression levels within diverse breast cancer cell lines, including the human triple-negative breast cancer cell lines BT-549, MDA-MB-231 and MDA-MB-468, the luminal A breast cancer cell lines T47D and MCF-7, and the mouse breast cancer cell line 4T1, and non-cancerous mammary epithelial cell line MCF-10 A as control for comparison. The real-time qPCR results indicated that SENP2 transcription in most breast cancer cell lines was detectable in these cell lines, with the highest level detected in MDA-MB-231 cells (Fig. 1A). Subsequent Western blotting analysis corroborated that these cell lines also expressed high levels of SENP2 protein, with the highest levels detected in BT-549 and MDA-MB-231 cells (Fig. 1B). This confluence of results pronounced high expression of both SENP2 mRNA and protein within breast cancer cells.

To unveil the clinical ramifications of SENP2 expression, we analyzed the expression level of SENP2 in breast cancer patients with the GEPIA2 database (quartile cutoff). The results showed that SENP2 was prominently expressed in breast cancer tissue in contrast to adjacent normal tissue (Fig. 1C). SENP2 was also highly expressed in luminal A, luminal B, and basal-like breast cancer tissues but not in HER2-positive breast cancer tissues (Fig. 1D). Although the data did not show statistically significant difference, an upward trend in SENP2 expression was observed in tumor tissues. Subsequently, we conducted an in-depth analysis of SENP2 expression using 10 case samples sourced from breast cancer patients and tissue chips derived from both cancerous and paracancerous tissues of breast carcinoma patients. Our results revealed a significant elevation in SENP2 expression within breast cancer tissue cells, characterized by an H-score value substantially surpassing that in corresponding adjacent tissues (Fig. 1E and F, and S1), and SENP2 expression was highly correlated with the stages of breast cancer progression (Fig. 1F), indicating that SENP2 was up-regulated in breast cancer tissues. Furthermore, in an endeavor to decipher the clinical implications, we harnessed RNA-seq data from the TCGA dataset with Kaplan-Meier survival analysis to elucidate the correlation between SENP2 expression and survival in breast cancer patients. Notably, our findings unveiled a significant inverse relationship, with heightened SENP2 expression levels being closely tied to a diminished overall survival rate (Fig. 1G), indicating that SENP2 overexpression is closely correlated with a poorer prognosis.

### SENP2 promotes breast cancer cell growth and tumorigenesis

**Fig. 2:**
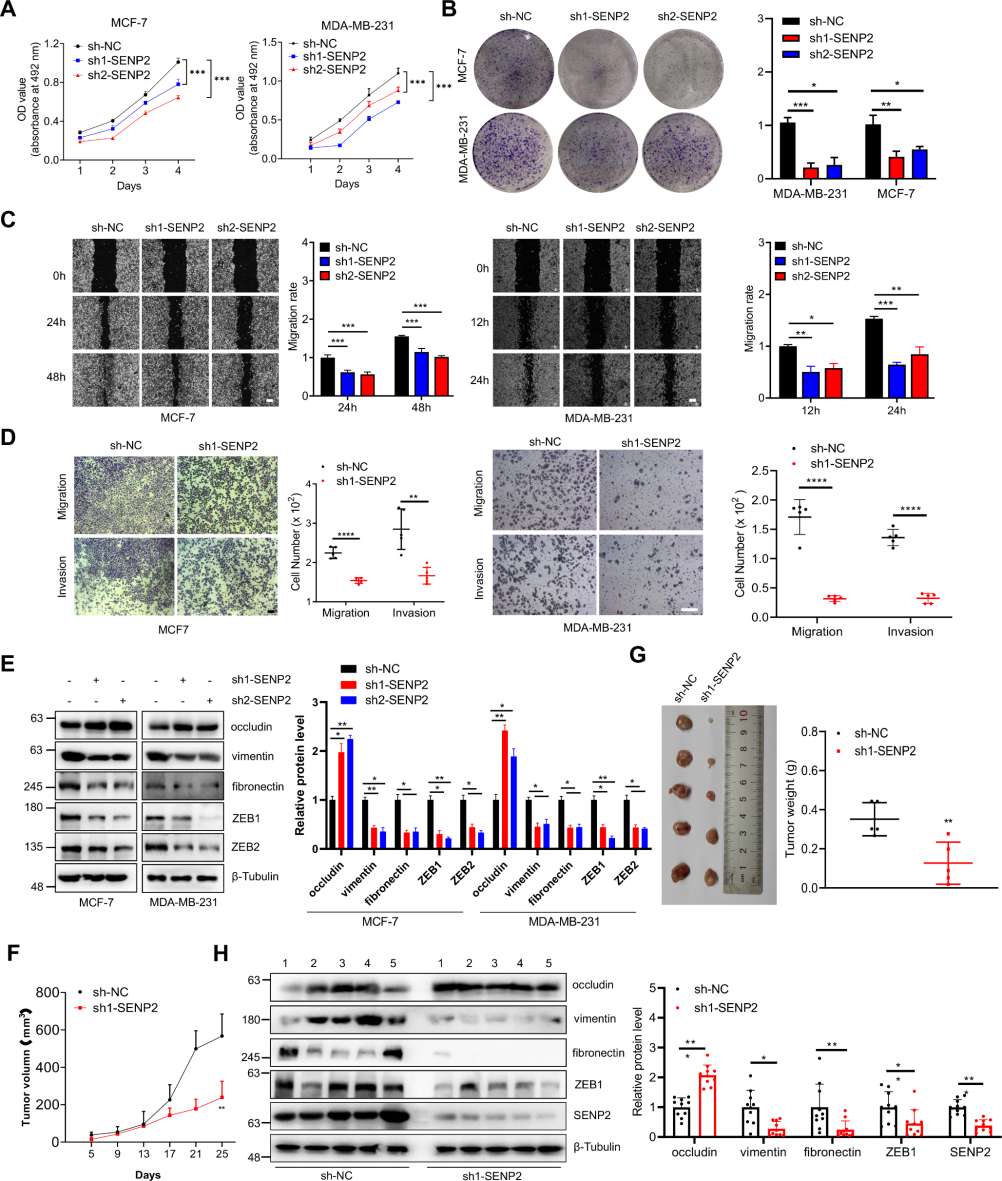
SENP2 knockdown inhibits the progression of breast cancer in vitro and in vivo. **A**. SENP2 knockdown inhibited the proliferation of MCF-7 and MDA-MB-231 cells. Cell growth curves of the SENP2 knockdown and control groups were generated from daily quantification of cell numbers (*n* = 3 biological repeats/group). **B**. SENP2 knockdown inhibited the colony formation of MCF-7 and MDA-MB-231 cells. Cells were seeded in 24-well plates for 14 days and stained with 0.1% crystal violet for the colony formation assay (left). The colony formation ability was analyzed (*n* = 3 biological repeats/group). **C**. SENP2 knockdown inhibited the migration of MCF-7 and MDA-MB-231 cells. Cell migration was detected by wound healing assay in stably transfected cells (left). The migration rate was measured and analyzed (right, *n* = 3 biological repeats/group). The scale bar is 200 μm. **D**. SENP2 knockdown inhibited the invasion of MCF-7 and MDA-MB-231 cells. Cell migration and invasion were analyzed by transwell assay in stably transfected cells (left). The migration and invasion of cells were measured and analyzed (right, *n* = 5 biological repeats/group). The scale bar is 200 μm. **E**. SENP2 knockdown inhibited EMT in MCF-7 and MDA-MB-231 cells. The sh-NC and two sh-SENP2 plasmids were packaged and transduced into cells, and the whole cell lysates were detected by Western blotting with the indicated antibodies (left). The results of the quantitative analysis of Western blotting are shown in the right panel (*n* = 3 biological repeats/group). **F**-**G**. SENP2 knockdown inhibited the tumorigenesis of breast cancer in vivo. The tumor volume (F) was measured every four days, and the tumors were harvested and weighed at the end of the experiment (G, *n* = 5 mice/group). **H**. SENP2 knockdown inhibited EMT in vivo. The breast tumor extracts from the sh-NC and sh1-SENP2 mice were analyzed by Western blotting with the indicated antibodies (left). The results of the quantitative analysis of Western blotting are shown in the right panel (*n* = 5 mice with 2 technical repeats/group)

In pursuit of a more profound understanding of SENP2 in breast cancer cells, the SENP2 gene was knocked down by two short hairpin RNAs (shRNAs) in both MCF-7 and MDA-MB-231 cells (Figures S2A and S2B). Remarkably, SENP2 knockdown significantly inhibited the growth of MDA-MB-231 and MCF-7 cells, whereas exogenous overexpression of SENP2 significantly enhanced the growth of tumor cells (Fig. 2A, S3A-S3C). Additionally, we delved into the realm of colony formation, where SENP2 knockdown led to a remarkable reduction in the colony formation ability of these cells, while SENP2 overexpression exerted the opposite effect (Fig. 2B and S3D). Expanding our horizons to encompass cellular mobility, SENP2 knockdown emerged as a potent suppressor of both migration and invasion capabilities in MCF-7 and MDA-MB-231 cells. In contrast, the overexpression of SENP2 conferred a marked promotion of migration and invasion in these tumor cells (Fig. 2C and D, S3E, and S3F), collectively underscoring the pivotal role played by SENP2 in contributing to breast cancer tumorigenesis. Furthermore, we embarked on an exploration of the EMT process affected by SENP2. The outcomes revealed that SENP2 knockdown decreased epithelial markers and increased mesenchymal markers, and vice versa by SENP2 overexpression in both MCF-7 and MDA-MB-231 cells (Fig. 2E, S2C, and S3G).

Subsequently, we extended our inquiry to in vivo models by examining the impact of SENP2 knockdown on the tumorigenesis of MDA-MB-231 cells in SCID mice. Notably, SENP2 knockdown significantly curtailed the initiation and progression of subcutaneous xenografts in these mice, as evidenced by the growth curve and tumor weight (Fig. 2F and G). Additionally, there was no significant difference in body weight between these two groups (Figure S2D). Further experiments showed that SENP2 also promoted the EMT process in vivo (Fig. 2H). These results indicate that SENP2 plays a pivotal role in the promotion of carcinogenesis in breast cancer.

### SENP2 regulates breast cancer cells in an ERK2-dependent manner

**Fig. 3:**
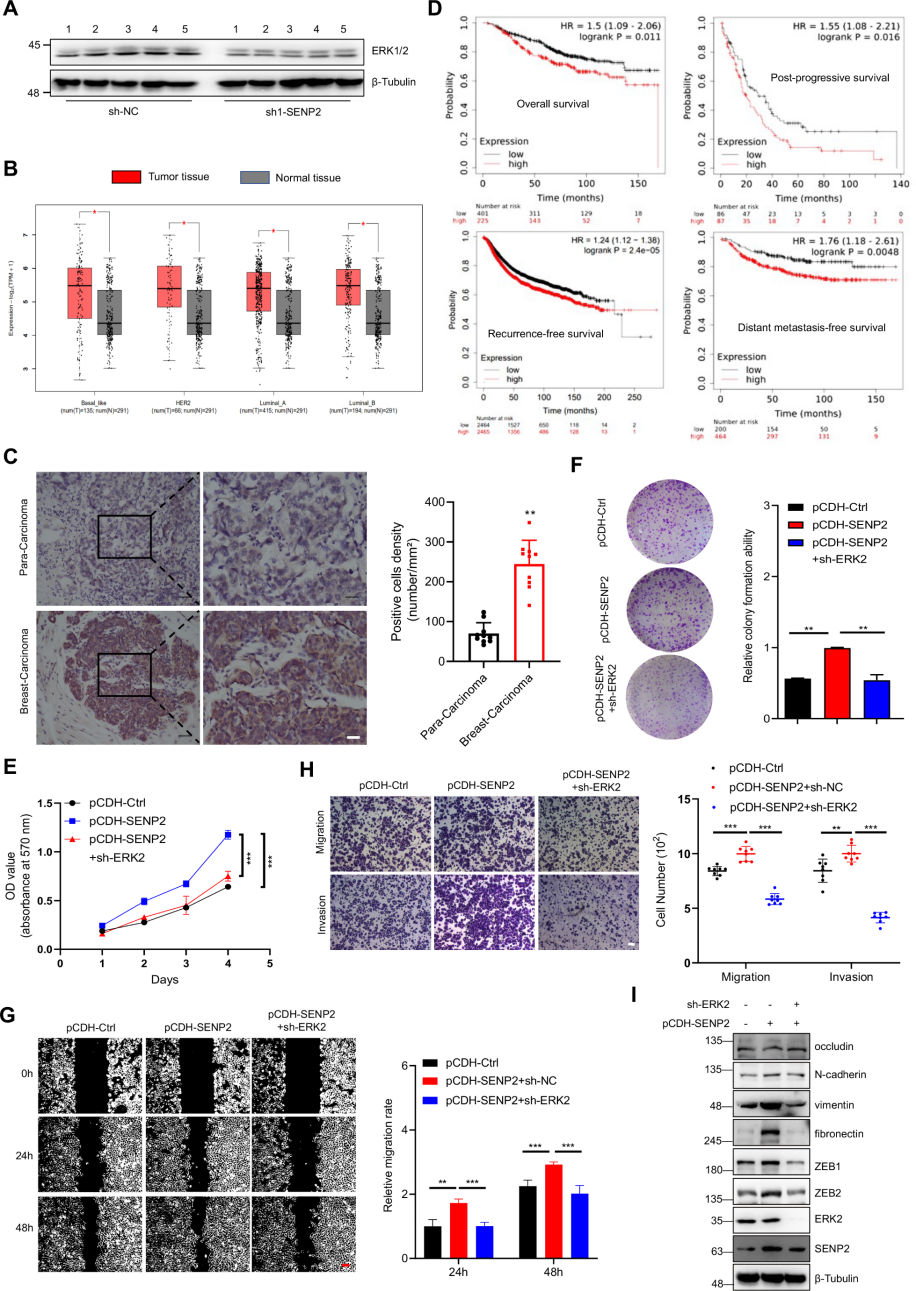
SENP2 promotes cell proliferation and migration depending on ERK2 in breast cancer cells. **A**. ERK2, but not ERK1, was significantly decreased in breast cancer tissue from SENP2 knockdown mice. The breast tumor extracts from the sh-NC and sh1-SENP2 mice were analyzed by Western blotting with the indicated antibodies. **B**. ERK2 was highly expressed in different subtypes of breast cancer tissues. ERK2 expression levels in various subtypes of breast cancer tissues were analyzed with the GEPIA2 database. **C**. ERK2 was highly expressed in breast cancer patients. ERK2 in breast cancer and precancerous tissue from breast cancer patients was detected by immunohistochemistry with the anti-ERK2 antibody (*n* = 10 biological repeats/group). Hematoxylin (blue) was applied to show the nuclei. The scale bar is 50 μm. **D**. ERK2 was highly correlated with distant metastasis-free survival, post-progression survival, and overall survival in breast cancer patients. The relationship between ERK2 expression and survival time in breast cancer patients was analyzed by survival analysis with the Kaplan-Meier Plotter database. **E**. The overexpression of SENP2 in MDA-MB-231 cells induced proliferation, a phenomenon that was subsequently rescued by the knockdown of ERK2. Growth curves for the specified cell groups were meticulously generated through daily cell number quantification (*n* = 3 biological replicates per group). **F**. ERK2 knockdown rescued the colony formation induced by SENP2 overexpression in MDA-MB-231 cells. Cells were seeded for 14 days and stained with 0.1% crystal violet for the colony formation assay (left). The colony formation ability was analyzed (*n* = 3 biological repeats/group). **G**. ERK2 knockdown rescued the migration induced by SENP2 overexpression in MDA-MB-231 cells. Cell migration was determined by wound healing assay in stably transfected cells (left). The migration rate was measured and analyzed (right, *n* = 3 biological repeats/group). The scale bar is 200 μm. **H**. ERK2 knockdown rescued the migration and invasion that was induced by SENP2 overexpression in MDA-MB-231 cells. Cell migration and invasion were analyzed by transwell assay in stably transfected cells (left). The migration and invasion of cells were measured and analyzed (right, *n* = 8 biological repeats/group). The scale bar is 200 μm. **I**. ERK2 knockdown rescued the EMT process promoted by SENP2 overexpression in MDA-MB-231 cells. The whole cell lysates were analyzed by Western blotting with the indicated antibodies

ERK, an established key orchestrator of EMT in breast cancer development, has been documented as an essential coordinator [31]. ERK2 activity and its downstream effects can be influenced by various types of post-translational modifications, including phosphorylation, ubiquitination, and S-acylation [32–34]. It is unknown whether ERK2 is regulated by SENP2-mediated deconjugation of SUMOylation. Our investigation unveiled a significant decline in the protein levels of ERK2, but not ERK1, following SENP2 knockdown in the xenograft mouse model (Fig. 3A), suggesting that SENP2 specifically regulated ERK2 but not ERK1 in breast cancer. To shed light on this enigma, we conducted an extensive analysis of ERK expression levels in breast cancer patients utilizing the GEPIA2 database (quartile cutoff), and the outcomes revealed that ERK2 but not ERK1 was prominently upregulated in four subtypes of breast cancer tissues (Fig. 3B and S4A). Furthermore, our analysis of ERK2 expression within patient-derived cancer and paracancerous tissues showed a substantial elevation in ERK2 levels within breast cancer tissue cells, with the H-score value significantly exceeding that in corresponding adjacent tissues (Fig. 3C). Furthermore, we harnessed the wealth of RNA-seq data housed in the TCGA dataset and conducted Kaplan-Meier survival analysis to delve into the intricate correlation between ERK2 expression levels and the survival of breast cancer patients. Astonishingly, our findings illuminated a profound and adverse association. Patients exhibiting high ERK2 expression levels were closely aligned with diminished overall survival, post-progressive survival, recurrence-free survival, and distant metastasis-free survival (Fig. 3D). Further analysis showed that high ERK2 expression levels were highly correlated with decreased overall survival of patients with TNBC and luminal A breast cancer (Figure S4B), suggesting that ERK2 overexpression is correlated with a poorer prognosis of subtypes of breast cancer.

Our exploration prompted the investigation of the influence of ERK2 on breast cancer cells by successfully knocking down ERK2 expression with shRNA in MDA-MB-231 and MCF-7 cells (Figure S4C). ERK2 knockdown manifested as a potent inhibitor of MDA-MB-231 and MCF-7 cell growth (Figure S4D). Additionally, the colony formation ability was significantly weakened after ERK2 knockdown (Figure S4E). Furthermore, ERK2 suppression dramatically suppressed the migration and invasion of MDA-MB-231 and MCF-7 cells (Figures S4F and S4G), indicating that ERK2 contributed to the tumorigenesis of breast cancer. Moreover, we assessed whether the EMT process was affected by ERK2. The results showed that ERK2 knockdown significantly increased epithelial markers and decreased mesenchymal markers in MDA-MB-231 and MCF-7 cells (Figure S4H).

The results implicated important role of ERK2 in breast cancer pathogenesis and indicated a potential crosstalk between SENP 2 and ERK2. Our results revealed that the suppression of ERK2 effectively rescued the enhanced colony formation ability, migration, and EMT process of cancer cells triggered by SENP2 overexpression in MDA-MB-231 cells (Fig. 3E and I). Additionally, the rescue assay indicated that ERK2 overexpression significantly activated the inhibited colony formation ability, migration, and EMT process of cancer cells induced by SENP2 knockdown (Figures S4H-S4K), indicating that SENP2 propels the progression of breast cancer by upregulating the level of ERK2.

### SUMO2 and SENP2 coordinate the SUMOylation of ERK2

**Fig. 4:**
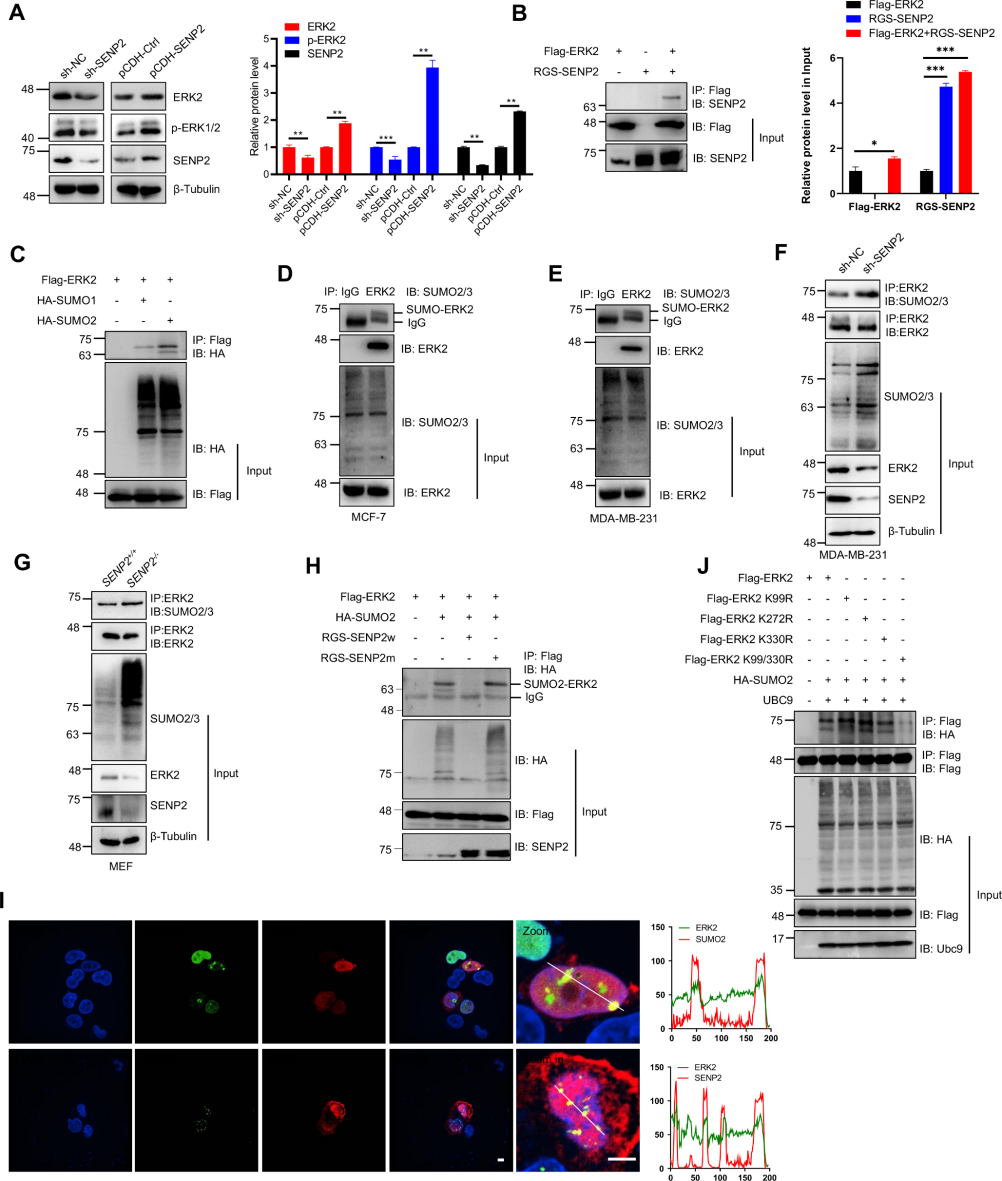
ERK2 is SUMOylated by SUMO2 and deSUMOylated by SENP2. **A**. The protein level of ERK2 was decreased in the SENP2-engineered breast cancer cells. The whole cell lysates of SENP2 knockdown MDA-MD-231 (left) and overexpressed MCF7 (middle) cells were analyzed by Western blotting with the indicated antibodies. The quantitative analysis of Western blotting results is displayed in the right panel (*n* = 3 biological replicates/ group). **B**. SENP2 interacted with ERK2. The indicated plasmids were transfected into HEK293T cells, and the IP with anti-Flag from whole cell lysates was detected by Western blotting with anti-SENP2 antibody. The whole cell lysates were detected by Western blotting with anti-Flag and anti-SENP2 antibodies (left). The quantitative analysis of Western blotting results is displayed in the right panel (*n* = 3 biological replicates/ group). **C**. ERK2 is predominantly modified by exogenous SUMO2 within HEK293T cells. The designated plasmids were transfected into the HEK293T cells, and the IP using anti-Flag from whole cell lysates was detected via Western blotting with anti-HA antibody. The whole cell lysates were detected by Western blotting with anti-HA or anti-Flag antibodies. **D**-**E**. ERK2 was mainly modified by endogenous SUMO in MCF7 (D) and MDA-MB-231 (E) cells. The IP with anti-IgG or anti-ERK2 from whole cell lysates was detected by Western blotting with the indicated antibodies. The WCL was detected by Western blotting with the indicated antibodies. **F**-**G**. SENP2 knockdown and knockout enhanced the endogenous SUMOylation of ERK2. The IP with anti-ERK2 from whole cell lysates of SENP2 knockdown MDA-MB-231 cells (G) and SENP2 knockout MEF cells (G) was detected by Western blotting with the indicated antibodies. The WCL was detected by Western blotting with the indicated antibodies. **H**. SENP2 catalyzed the deconjugation of ERK2 SUMOylation within HEK293T cells. The specified plasmids were transfected into HEK293T cells, and the IP with anti-Flag from cell lysates was detected by Western blotting with anti-HA antibody. The WCL was detected through Western blotting with the indicated antibodies. **I**. ERK2 was co-localized with SUMO2 and SENP2 in the nucleus and cytoplasm. The Flag-ERK2 plasmid was transfected into HEK293T cells, and the cells were harvested for immunofluorescence staining with anti-Flag (red) and anti-SUMO2 (green) or anti-SENP2 (green) antibodies. DAPI (blue) was used to show nuclei, and the colocalization curve was analyzed by ImageJ software. The scale bar is 5 μm. **J**. K99 and K330 were the primary SUMOylation sites of ERK2. Wild-type (w) or SUMO site mutant Flag-ERK2 and HA-SUMO2 were transiently transfected into HEK293T cells, and the IP with anti-Flag from cell lysates was detected by Western blotting with anti-HA and anti-Flag antibodies. The WCL was detected by Western blotting with anti-HA or anti-Flag antibodies

Our findings illuminate the specific regulatory role of SENP2 over ERK2 protein expression (Fig. 3A), and we next conducted a comprehensive examination of ERK2 expression in MDA-MB-231 cells engineered with varying SENP2 levels. Notably, our results revealed a highly correlated relationship between protein expression of ERK2 and phosphorylated ERK2 and SENP2 levels (Fig. 4A). Furthermore, we explored ERK2 expression in SENP2-deficient mouse embryonic fibroblast (MEF) cells and SENP2 knockdown MCF-7 cells, revealing a striking reduction in ERK2 protein levels, despite comparable ERK2 mRNA levels between SENP2^−/−^ and SENP2^+/+^ MEF cells (Figures S5A-S5C). Further experiments showed that SENP2 specifically interacted with ERK2 (Fig. 4B), and the interacted SENP2 regulates the protein but not transcriptional level of ERK2, indicating that SENP2 regulates ERK2 at the post-translational level.

Intriguingly, our computational analysis, utilizing the SUMOplot database (www.abcepta.com/sumoplot), pinpointed ERK2 as a prime candidate for SUMOylation (Figure S5C). Subsequent co-immunoprecipitation (co-IP) assays in HEK293T cells revealed that ERK2 predominantly forms conjugates with exogenous SUMO2 (Fig. 4C). Then the co-IP results showed that ERK2 was also SUMOylated by endogenous SUMO2 in MCF-7 and MDA-MB-231 cells (Fig. 4D and E). Furthermore, the endogenous SUMOylation levels of ERK2 were significantly increased in SENP2 knockdown MDA-MB-231 cells and SENP2 deficiency MEF cells (Fig. 4F and G). The reversible nature of SUMOylation, mediated by members of the SENP family, prompted us to conduct deconjugation experiments. Notably, wild-type SENP2, but not a catalytic mutation of SENP2, demonstrated the ability to effectively deconjugate SUMOylated ERK2 (Fig. 4H). IF staining revealed that ERK2 co-localized with endogenous SUMO2 and SENP2 in the nuclei of HEK293T cells (Fig. 4I).

As we probed deeper into the SUMOylation landscape of ERK2, bioinformatics analysis identified two potential consensus sites for SUMO conjugation, notably K99 and K330 (Figure S5D). These predicted SUMO sites displayed a high degree of evolutionary conservation across diverse species (Figure S5E). Subsequently, our endeavors to pinpoint ERK2 SUMOylation sites involved the generation of ERK2 plasmid constructs, including those with single or double SUMOylation site mutations. The mapping results conclusively identified K99 and K330 as the primary SUMOylation sites within ERK2 (Fig. 4J). Taken together, these results indicate that ERK2 is SUMOylated by SUMO2 and that SENP2 interacts with ERK2 to deconjugate its SUMOylation.

### ERK2 SUMOylation inhibits breast cancer cell growth and tumorigenesis

**Fig. 5:**
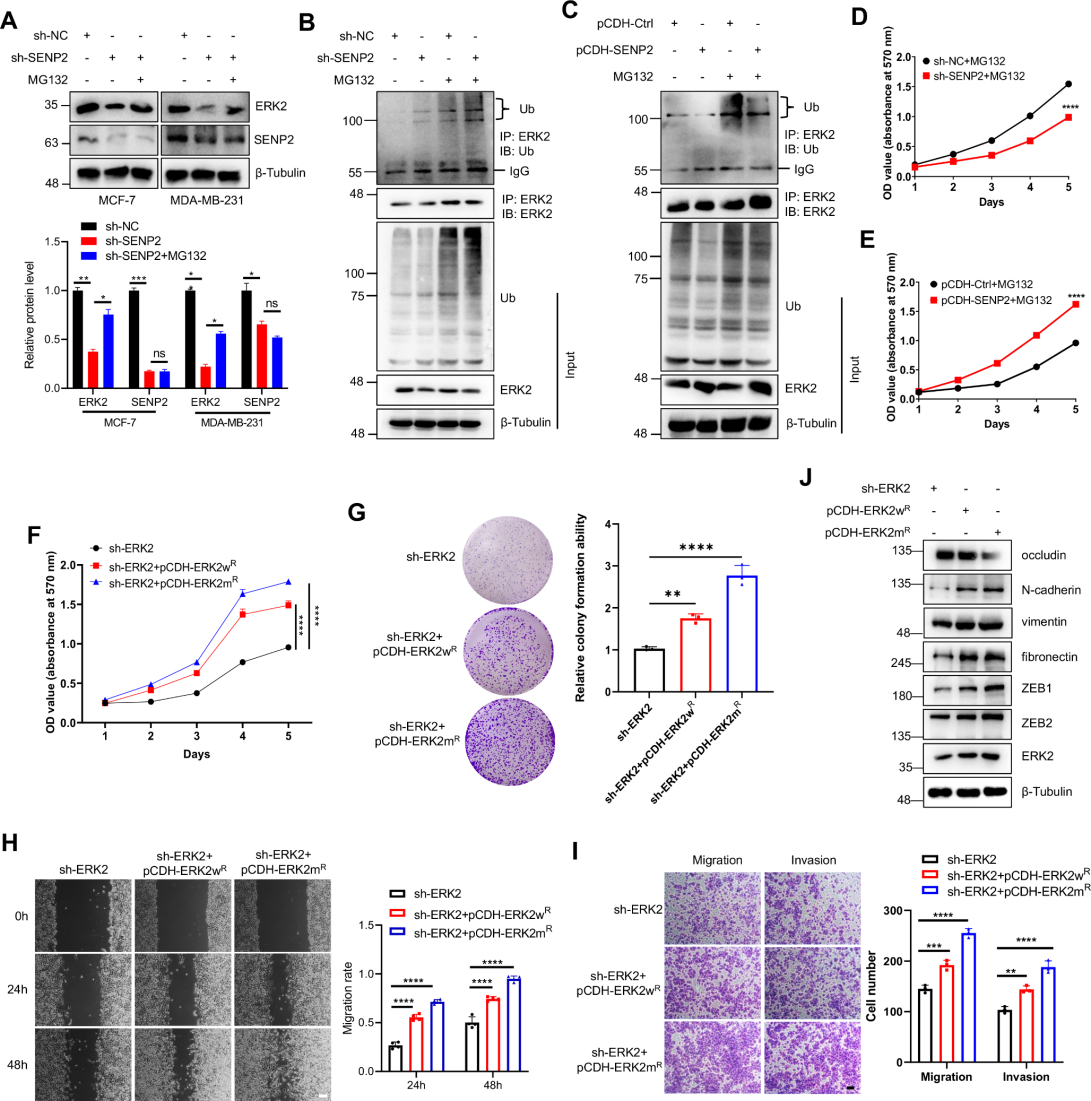
SUMO site mutant ERK2 enhanced the increased cell growth and metastasis induced by wild-type ERK2. **A**. The decreased expression of ERK2 in breast cancer cells was rescued by MG132 treatment. MDA-MB-231 and MCF-7 cells were treated with MG132, and the WCL were harvested and detected by Western blotting with the indicated antibodies (upper panel). The quantitative analysis of Western blotting results is displayed in the bottom panel (*n* = 3 biological replicates/ group).**B**-**C**. SENP2 accelerated the ubiquitination of the endogenous ERK2 protein. Cells were treated with DMSO or MG132, and the IP with anti-ERK2 from cell lysates of SENP2 knockdown (B) or overexpressing (C) MDA-MB-231 cells were detected by Western blotting with anti-ubiquitin or anti-ERK2 antibodies. The WCL was detected through immunoblotting using the designated antibodies.**D**-**E**. SENP2 promoted the proliferation of MDA-MB-231 cells. Cells were treated with DMSO or MG132, and the growth curves of SENP2 knockdown (D) or overexpressing (E) MDA-MB-231 cells were generated from daily quantification of cell numbers (*n* = 3 biological repeats/group).**F**. SUMO mutant and shRNA resistant ERK2 enhanced the proliferation of MDA-MB-231 cells. Growth curves of wild-type or SUMO mutant ERK2 cells were generated from daily quantification of cell numbers (*n* = 3 biological repeats/group).**G**. SUMO mutant and shRNA resistant ERK2 enhanced the colony formation of MDA-MB-231 cells. Cells were seeded for 14 days and stained with 0.1% crystal violet for the colony formation assay (left). The colony formation ability was analyzed (*n* = 3 biological repeats/group).**H**. SUMO mutant and shRNA resistant ERK2 enhanced the migration of MDA-MB-231 cells. Cell migration was detected by wound healing assay in stably transfected cells (left). The migration rate was measured and analyzed (right, *n* = 4 repeats/group). The scale bar is 200 μm. **I**. SUMO mutant and shRNA resistant ERK2 enhanced the invasion of MDA-MB-231 cells. Cellular invasiveness was assessed via the transwell assay in cells stably transfected with the construct (left). The invasive cells were measured and analyzed (right, *n* = 3 biological repeats/group). The scale bar represents 200 μm. **J**. SUMOylation of ERK2 regulates the EMT process in breast cancer cells. Wild-type or SUMOylation site mutant and shRNA resistant ERK2 was stably expressed in MDA-MB-231 cells, and whole cell lysates were detected by Western blotting with the indicated antibodies

Notably, our previous findings illuminate the specific regulatory role of SENP2 over ERK2 protein expression in xenograft mice model (Fig. 3A) and MDA-MB-231 cells (Fig. 4A). To delve into the nuanced interplay of PTM and their impact on ERK2, we turned to MG132 treatment to inhibit the ubiquitin-dependent degradation pathway within breast cancer cells. The outcomes demonstrated the pivotal role of SUMOylation in orchestrating ERK2 stability, as MG132 treatment effectively shielded ERK2 from degradation in both breast cancer cells (Fig. 5A), suggesting that SUMOylation regulates ERK2 stability via the ubiquitin-proteasome degradation pathway. Furthermore, we detected the extent of endogenous ubiquitination of ERK2 in MDA-MB-231 cells and identified that SENP2 reduction was associated with the promotion of ERK2 degradation, whereas SENP2 overexpression inhibited its degradation (Fig. 5B and C). Further experiments showed that SENP2 reduction promoted ERK2 degradation and inhibited cell proliferation, while SENP2 overexpression inhibited ERK2 degradation and promoted cell proliferation (Fig. 5D and E). MG132 is a well-established proteasome inhibitor which prevents protein degradation via the ubiquitin-proteasome pathway. This inhibition leads to the accumulation of ERK2, allowing it to remain signaling active for a longer period of time than normal. To identify the effect of ERK2 SUMOylation on breast cancer cells, we constructed rescued stable MDA-MB-231 cells by lentiviral transduction of ERK2 knockdown cells with wild-type or SUMO site mutant ERK2 plasmids that were resistant to ERK2 shRNA. We first assessed the role of wild-type or mutant ERK2 in cell growth, which showed that mutant ERK2 significantly promoted cancer cell growth (Fig. 5F). In addition, these cells displayed significantly enhanced colony formation capacity (Fig. 5G), indicating that ERK2 SUMOylation suppressed the proliferation of breast cancer cells. Furthermore, mutant ERK2 dramatically increased the migration and invasion of MDA-MB-231 cells (Fig. 5H and I), indicating that blockade of ERK2 SUMOylation contributed to tumorigenesis of breast cancer. To shed light on the intricate relationship between ERK2 SUMOylation and the EMT process in breast cancer cells, we overexpressed either wild-type or mutant ERK2 in MDA-MB-231 cells. Strikingly, the introduction of the SUMOylation mutant ERK2 led to a remarkable increase in the EMT-associated mesenchymal markers (Fig. 5J). To confirm the effect of ERK2 SUMOylation on breast cancer cells, we constructed stable MDA-MB-231 cells by lentiviral transduction with wild-type or SUMO site mutant ERK2 plasmids, and the experiments showed similar results (Figures S6A-S6E). These results indicated that blockade of ERK2 SUMOylation promotes the EMT process and ultimately contributes to the tumorigenesis of breast cancer cells.

### MiR-145-5p is a direct upstream regulator of SENP2 in breast cancer cells

**Fig. 6:**
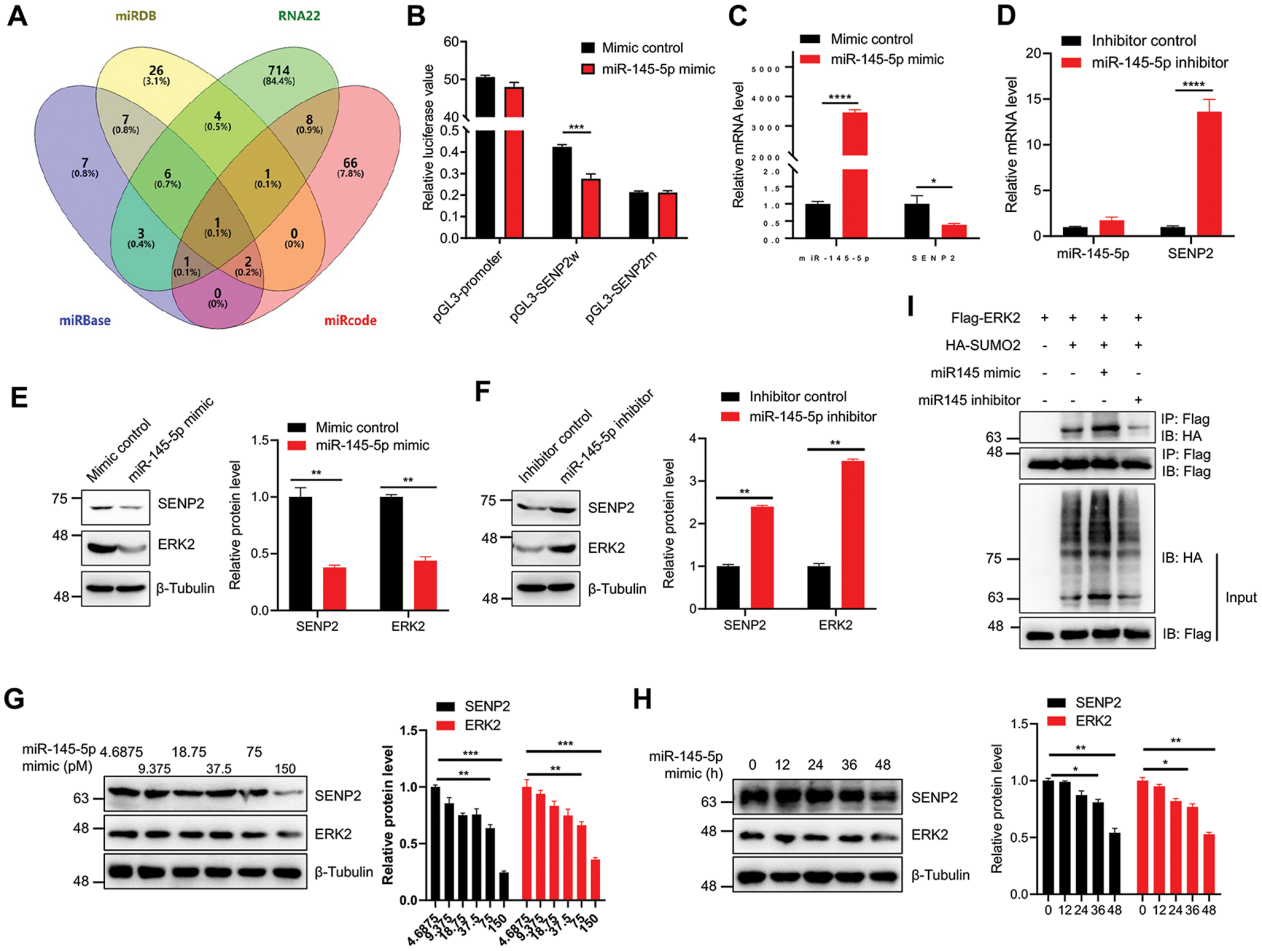
miR-145-5p is a direct upstream regulator of SENP2 in breast cancer cells. A. miR-145-5p is predicted to be an upstream regulator of SENP2. The potential upstream microRNA that regulates SENP2 transcription was predicted by miRDB, miRBase, miRcode, and RNA22. **B**. The miR-145-5p mimic inhibited the promoter activity of wild-type but not mutant SENP2. HEK293T cells were transiently transfected with the wild-type mutant SENP2 promoter, and relative luciferase activity was detected after treatment with the miR-145-5p mimic or control (*n* = 3 biological repeats/group). **C**. miR-145-5p mimic inhibited the transcription of SENP2. MDA-MB-231 cells were treated with miR-145-5p mimic or control, and the expression level of SENP2 transcripts was calculated by real-time PCR and normalized to the control (*n* = 3 biological repeats/group). **D**. Inhibition of miR-145-5p resulted in the upregulation of SENP2 transcription. MDA-MB-231 cells were treated with either a miR-145-5p inhibitor or a control, and the transcription level of SENP2 were determined by real-time PCR and normalized to the control group (*n* = 3 biological repeats/group). **E**. The miR-145-5p mimic inhibited the protein levels of SENP2 and ERK2. MDA-MB-231 cells were treated with miR-145-5p mimic or control, and the protein expression levels of SENP2 and ERK2 were measured by Western blotting with the indicated antibodies (left). The quantitative analysis of Western blotting results is displayed in the right panel (*n* = 3 biological replicates/group). **F**. The miR-145-5p inhibitor promoted the protein expression of SENP2 and ERK2. MDA-MB-231 cells were treated with miR-145-5p inhibitor or control, and the protein expression levels of SENP2 and ERK2 were measured by Western blotting with the indicated antibodies (left). The quantitative analysis of Western blotting results is displayed in the right panel (*n* = 3 biological replicates/group). **G**-**H**. The miR-145-5p mimic inhibited the level of SENP2 and ERK2 protein in a concentration (G) and time-dependent (H) manner. MDA-MB-231 cells were treated with miR-145-5p mimic for different concentrations and times, and the protein expression levels of SENP2 and ERK2 were measured by IB with the indicated antibodies (left). The quantitative analysis of Western blotting results is displayed in the right panel (*n* = 3 biological replicates/group). **I**. MiR-145-5p enhanced the SUMOylation level of ERK2. The specified plasmids were transiently transfected into HEK293T cells, followed by IP using anti-Flag antibodies from cellular lysates, and subsequent Western blotting was performed using anti-HA antibodies. The WCL was detected by Western blotting with anti-HA and anti-Flag antibodies

SENP2 deSUMOylates numerous proteins to regulate their functions; however, the regulation of SENP2 per se remains unclear. Previous studies have underscored the effect of microRNAs (miRNAs) on modulating SENP family members in various cancer contexts [35–39]. To identify the upstream microRNA that regulates SENP2 in breast cancer, we used the miRBase, miRDB, RNA22, and miRcode databases to predict the potential upstream regulatory factors. All data with a prediction score of less than 0.8, poor species conservation, or a *p* value of less than 0.05 were removed from the respective database to obtain the intersection of the remaining data among these four databases. The analysis indicated that miR-145-5p was the only predicted regulator of SENP2 (Fig. 6A). The miRBase database was then applied to predict the specific binding sequence between miR-145-5p and SENP2, showing that miR-145-5p interacted primarily with the 804–810 sites of the 3’-UTR of SENP2 mRNA (Figure S6A), which is evolutionarily highly conserved among different species (Figure S6B).

To ascertain the regulatory impact of miR-145-5p on SENP2 transcription, the wild-type or mutant 3’-UTR fragment of SENP2 was cloned and inserted into the pGL3-promoter luciferase reporter vector (Figure S6B). Notably, the miR-145-5p mimic significantly reduced luciferase activity specifically within the wild-type 3’-UTR of SENP2 in HEK293T cells, affirming direct targeting by miR-145-5p (Fig. 6B), indicating that miR-145-5p directly targeted the 3’-UTR of SENP2. Moreover, the miR-145-5p mimic elicited a noteworthy decrease in SENP2 mRNA levels, while the miR-145-5p inhibitor increased the level of SENP2 mRNA in MDA-MB-231 cells (Fig. 6C and D). The SENP2 and ERK2 protein levels were downregulated by the miR-145-5p mimic and upregulated via the inhibitor of miR-145-5p (Fig. 6E and F), and the miR-145-5p mimic repressed the expression level of SENP2 and ERK2 protein in a concentration and time-dependent manner (Fig. 6G and H). Since SENP2 deSUMOylated ERK2, we further assessed ERK2 SUMOylation by the miR-145-5p mimic and inhibitor in HEK293T cells. The results unequivocally indicated that ERK2 SUMOylation was elevated by the miR-145-5p mimic and weakened by the miR-145-5p inhibitor (Fig. 6I), indicating that miR-145-5p down-regulates the SENP2-ERK2 axis in breast cancer cells.

### Mir-145-5p inhibits breast cancer cell growth and tumorigenesis in a SENP2-dependent manner

**Fig. 7:**
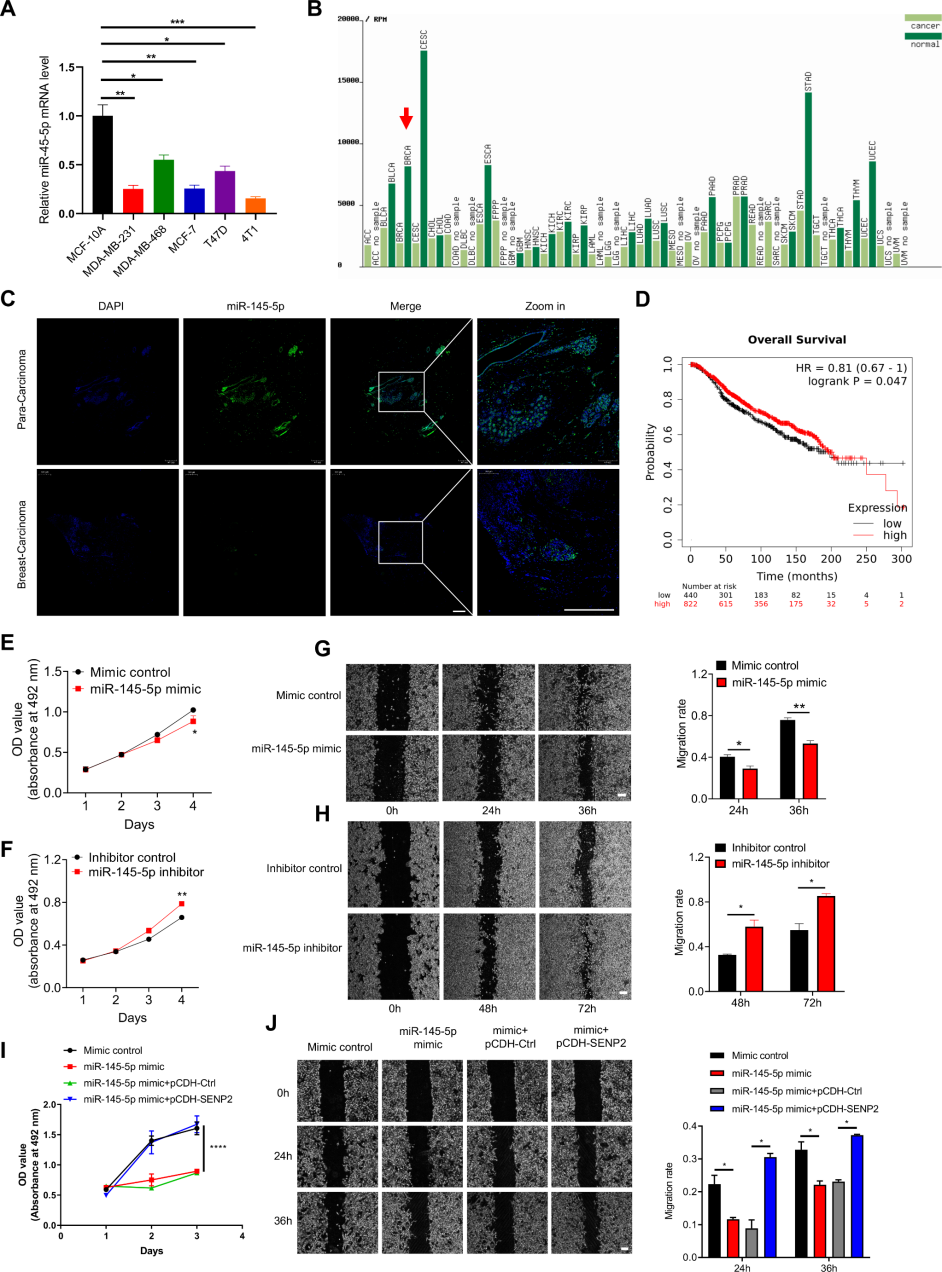
miR-145-5p acts as a tumor suppressor in breast cancer cells. **A**. Quantification of miR-145-5p transcript abundance across normal breast cancer cell MCF-10 A and diverse breast cancer cell lines. The transcript abundance of miR-145-5p in distinct breast cancer cell lines was assessed via real-time PCR and subsequently standardized to the expression levels in MDA-MB-231 cells (*n* = 3 biological repeats/group). **B**. miR-145-5p was poorly expressed in breast cancer tissue. miR-145-5p expression levels in various cancer tissues and normal tissues were analyzed with the Tumor-miRNA-Pathway database. **C**. miR-145-5p was poorly expressed in breast cancer patients. miR-145-5p in breast cancer and precancerous tissue from TNBC patients was analyzed by immunofluorescence staining with the miR-145-5p probe. DAPI (blue) was applied to label the nuclei. The scale bar is 50 μm. **D**. miR-145-5p was positively correlated with the overall survival of breast cancer patients. The relationship between miR-145-5p expression and survival rate in breast cancer patients was analyzed by survival analysis with the Kaplan-Meier Plotter database. **E**. The miR-145-5p mimic inhibited the proliferation of MDA-MB-231 cells. MDA-MB-231 cells were treated with miR-145-5p mimic or control, and growth curves of cells were calculated from daily quantification of cell numbers (*n* = 3 biological repeats/group). **F**. The miR-145-5p inhibitor promoted the proliferation of MDA-MB-231 cells. MDA-MB-231 cells were treated with miR-145-5p inhibitor or control, and cell growth curves were calculated from daily quantification of cell numbers (*n* = 3 biological repeats/group). **G**. miR-145-5p inhibited the migration of MDA-MB-231 cells. Cell migration was detected by wound healing assay in control or miR-145-5p mimic treated cells (left). The migration rate was calculated and analyzed (right, *n* = 3 biological repeats/group). The scale bar is 200 μm. **H**. The miR-145-5p inhibitor promoted the migration of MDA-MB-231 cells. Cell migration was detected by wound healing assay in control or miR-145-5p inhibitor-treated cells (left). The migration rate was calculated and analyzed (right, *n* = 3 biological repeats/group). The scale bar is 200 μm. **I**. SENP2 promoted the proliferation of MDA-MB-231 cells that were inhibited by the miR-145-5p mimic. SENP2 stably transfected MDA-MB-231 cells were treated with miR-145-5p mimic or control, and growth curves of cells were generated from daily quantification of cell numbers (*n* = 3 biological repeats/group). **J**. SENP2 promoted the migration of MDA-MB-231 cells that were inhibited by the miR-145-5p mimic. Cell migration was determined by wound healing assay in control or miR-145-5p inhibitor-treated cells (left). The migration rate was calculated and analyzed (right, *n* = 3 biological repeats/group). The scale bar is 200 μm

To identify the effect of miR-145-5p on the intricacies of breast cancer development, we conducted a comprehensive analysis of miR-145-5p expression levels in multiple breast cancer cell lines. The real-time qPCR results showed that the transcription of miR-145-5p was detectable in these cell lines, with the highest levels detected in MDA-MB-468 cells (Fig. 7A). We embarked upon a comparative exploration of the gene expression signatures associated with miR-145-5p in tumor and corresponding normal tissues with the Tumor-miRNA-Pathway database (http://bioinfo.life.hust.edu.cn/miR_path), and diminished miR-145-5p expression was evident in several tumor types, with breast cancer being a prominent exemplar (Fig. 7B). Moreover, we analyzed the expression of miR-145-5p by fluorescence in situ hybridization (FISH) using cancer and paracancerous tissue slides from patients with breast carcinoma. Our meticulous analysis illuminated a notable diminution in miR-145-5p levels within the breast cancer tissue cells, with the H-score exhibiting a significant decline in comparison to the corresponding adjacent tissues, indicating that miR-145-5p was down-regulated in breast cancer tissues (Fig. 7C). Furthermore, we conducted a wealth of RNA-sequencing data curated from the TCGA dataset with Kaplan-Meier survival analysis to elucidate the relationship between the expression level of miR-145-5p and the survival of patients with breast cancer. The results of this analysis were unequivocal: a strong positive correlation emerged between elevated levels of miR-145-5p and enhanced overall survival among patients afflicted with breast cancer (Fig. 7D), indicating that miR-145-5p overexpression is correlated with a better prognosis.

To identify the effect of miR-145-5p on breast cancer cells, we embarked on a transformative journey wherein MDA-MB-231 cells were treated with either a miR-145-5p mimic or inhibitor and detected the roles of miR-145-5p on cell growth and migration. We first evaluated the effect of the miR-145-5p mimic on cell growth, showing that the miR-145-5p mimic significantly inhibited cancer cell growth, whereas the miR-145-5p inhibitor promoted cancer cell growth (Fig. 7E and F). In addition, the miR-145-5p mimic dramatically suppressed the migration of MDA-MB-231 cells, while the miR-145-5p inhibitor significantly enhanced the migration of tumor cells (Fig. 7G and H), indicating that miR-145-5p inhibited the tumorigenesis of breast cancer. Furthermore, we detected the role of overexpressed SENP2 in miR-145-5p mimic transfected MDA-MB-231 cells. The results showed that overexpression of SENP2 rescued the inhibited cell growth and migration of cancer cells induced by the miR-145-5p mimic (Fig. 7I and J), substantiating the assertion that miR-145-5p operates as a pivotal agent in impeding the progression of breast cancer through down-regulating SENP2 levels.

## Discussion

Although the current clinical diagnostics and treatments for breast cancer are constantly updated, the cure rate is still generally low due to its malignancy [40]. Increasing evidence has shown that many proteins, such as α-catenin, AMPKα, and FOXM1B, are reported to be SUMOylated, which regulates the progression of breast cancer [41]. Previous studies have elucidated that SENP2 transcriptionally represses estrogen receptor alpha-regulated proliferation of MCF-7 cells [42]. Nevertheless, the molecular mechanism of SENP2 deconjugation of SUMOylation in breast cancer is largely unknown. Specifically, our studies have illuminated the propensity of SENP2 to enhance cell proliferation and facilitate cell migration and invasion both in vitro and in vivo (Fig. 2 and S2), which is consistent with the previous study of SENP2 in TGF-β-induced breast cancer [18], suggesting the important role of SENP2 in the tumorigenesis of breast cancer.

The Raf-MEK-ERK cascade serves as a conduit for mitogenic signaling and is frequently harnessed by Ras in the context of human cancers. Ras proteins are SUMOylated, and SUMOylation regulates their activation and facilitates cell migration and tumorigenesis [43]. Ras activates the ERK pathway and not only primes the ERK pathway through the activation of Raf but also orchestrates a pivotal role in inhibiting MEK SUMOylation, thus inducing carcinogenesis [44]. We identified that SENP2 performs its tumorigenic activity through the promotion of the EMT process in an ERK2-dependent manner (Fig. 3). Previous studies have underscored the pivotal role of ERK2, in contrast to ERK1, in inducing the EMT process and cancer stemness in breast epithelial cells [45], which is consistent with our findings. Furthermore, we identified that ERK2 is primarily SUMOylated by SUMO2 and deSUMOylated by SENP2 (Fig. 4). More importantly, mutation of the ERK2 SUMOylation sites further promoted cell growth, colony formation capacity, cell migration and invasion of breast cancer cells and tumorigenesis (Fig. 5), indicating the important role of ERK2 SUMOylation in the tumorigenesis of breast cancer.

The current study further identified a novel mechanism through which SENP2 regulates ERK2 stability via deSUMOylation (Figs. 4 and 5), a post-translational modification not extensively explored in breast cancer to date. ERK2 is a known mediator of cancer cell proliferation and survival [20], and this insight adds to the current understanding of the important role of ERK2 in oncogenic signaling of breast cancer. ERK2 was reported to be activated via lysine-63-linked polyubiquitination [33], and the current results showed that SENP2 deficiency induced hyper-SUMOylation of ERK2, thereby destabilizing ERK2 in ubiquitin-proteasome pathway (Fig. 5A and C). The current results showed that SUMOylation enhanced the polyubiquitination of ERK2, especially in the condition of MG132 treatment (Fig. 5B and C). These experiments indicated that potential regulator mediates SUMOylation-induced ubiquitination of ERK2, which needs further investigation.

SENP2 deconjugates multiple target proteins; however, the upstream regulator of SENP2 remains unclear. As short non-coding RNA, microRNA directly leads to mRNA degradation by binding to the 3’UTR of downstream target genes [46]. miR-145-5p was reported to regulate the development of breast cancer via several target genes [26, 47–49]. Our investigations have unveiled a significant revelation: miR-145-5p directly controls SENP2 transcription in breast cancer cells (Fig. 6). This discovery underscores a novel mechanistic dimension to the critical role of miR-145-5p in breast cancer. miR-145-5p directly bound to the 3’UTR of SENP2 mRNA to down-regulate the level of the SENP2 protein, thus promoting ERK2 SUMOylation and ultimately inhibiting the progression of breast cancer (Fig. 7). These findings highlight the critical role of the miR-145-5p and SENP2 axis, showing that SUMOylation protects cells by inhibiting the tumorigenesis of breast cancer. The current study investigated the critical role of miR-145-5p in the regulation of SENP2 deconjugated ERK2 SUMOylation, offering a unique perspective on the complex regulatory network that governs breast cancer pathogenesis, expanding upon the known roles of miR-145-5p in breast cancer [50].

ERK is essential for cellular processes, making ERK inhibitors potentially unsuitable due to collateral damage [51]. Our study proposes a novel strategy to selectively target cancer cells by modulating the SUMOylation status of ERK2. SUMOylation can alter protein stability and function in a context-dependent manner. By specifically targeting SENP2-mediated deSUMOylation of ERK2, we aim to modulate its activity selectively in cancer cells where SUMOylation machinery is dysregulated. Our data show that SENP2 is highly expressed in breast cancer tissues compared to normal tissues, providing an opportunity to selectively target the SENP2-ERK2 axis in cancer cells (Figs. 1, 2 and 3). Additionally, miR-145-5p downregulates SENP2 expression in breast cancer cells, enhancing ERK2 SUMOylation and selectively inhibiting cancer cell proliferation and metastasis (Figs. 4, 5, 6 and 7). This approach leverages the cancer-specific dysregulation of miR-145-5p to achieve selective targeting. Thus, our strategy exploits the dysregulated SUMOylation pathway and differential expression of SENP2 in cancer cells, holding promise for developing more effective and safer therapeutic interventions for breast cancer.

Our study has elucidated pivotal molecular mechanisms and potential therapeutic targets within the heterogeneous landscape of breast cancer. We unraveled the multifaceted role of SENP2 in breast cancer tumorigenesis. It is shown that SENP2 facilitates breast cancer progression both in *vitro* and in *vivo*, highlighting its importance as a potential biomarker and therapeutic target. Significantly, we discovered that SENP2 is involved in the post-translational modification of ERK2 through SUMOylation, which plays a critical role in stabilizing the protein and curbing EMT, a key process in cancer metastasis. Interestingly, we observed that the miR-145-5p targets SENP2 mRNA, revealing a novel layer of genetic regulation in this context. The ability of MiR-145-5p to downregulate SENP2 expression and boost ERK2 SUMOylation positions it as a promising candidate for therapeutic intervention to hinder breast cancer progression. However, the potential disparity between in *vitro* and in *vivo* environments needs further investigation. Therefore, future research should target the in vivo verification of these mechanisms, the exploration of these findings across diverse populations, and the synergistic potential of combination with existing treatments.

**Fig. 8:**
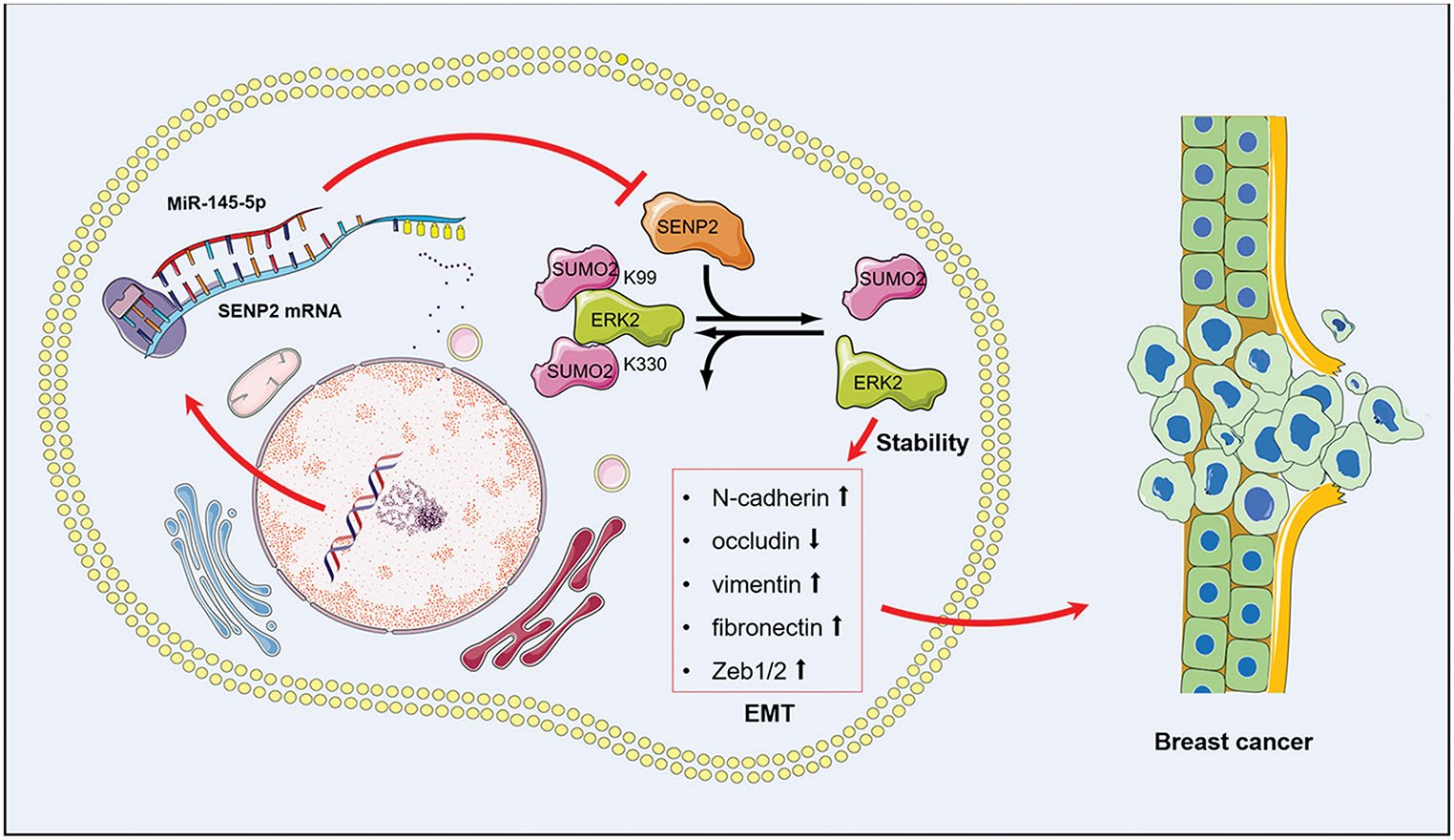
Schematic diagram of SENP2-deconjugated SUMOylation of ERK2 in miR-145-5p inhibited tumorigenesis of breast cancer. Firstly, miR-145-5p targets and binds to the 3’-UTR of SENP2 mRNA, resulting in the suppression of SENP2 expression; secondly, reduced SENP2 levels lead to the accumulation of SUMOylated ERK2, and SUMOylation of ERK2 favors its ubiquitination and proteasomal degradation; finally, degradation of ERK2 weakens the signaling pathways that promote EMT and tumorigenic processes in breast cancer cells

In summary, our data identified that ERK2 SUMOylation, mediated by the dynamic interplay of the miR-145-5p and SENP2 axis, is a critical determinant in the progression of breast cancer (Fig. 8). microRNA-145-5p inhibits SENP2 transcription, enhances SUMOylation of ERK2, and ultimately suppresses the progression of breast cancer. In conclusion, our studies suggest that the balance of ERK2 SUMOylation will be a novel intervention for breast cancer, providing an evolving therapeutic strategy for the clinical treatment of breast cancer.

## Electronic supplementary material

Below is the link to the electronic supplementary material.

Supplementary Material 1

## Data Availability

The authors confirm that the data supporting the findings of this study are available within the article and its supplementary materials, and all data are available from the corresponding author upon reasonable request.
