# Supplementary material for: MicroRNA-145-5p inhibits the tumorigenesis of breast cancer through SENP2-regulated ubiquitination of ERK2
Source: Cell Mol Life Sci. 2024 Nov 23;81(1):461. doi: 10.1007/s00018-024-05505-8 (PMC11584840; doi:10.1007/s00018-024-05505-8)
Supplement: Supplementary file 1 — Supplementary Material 1 [file 18_2024_5505_MOESM1_ESM.docx]

**Supplemental Tables**

**Table S1. Oligonucleotides.**

| Oligonucleotide | Sequence (5’-3’) |
| --- | --- |
| miR-145-5p probe | FAM-AGGGATTCCTGGGAAAACTGGAC-FAM |
| miR-145-5p mimic sense | GUCCAGUUUUCCCAGGAAUCCCU |
| miR-145-5p mimic antisense | GGAUUCCUGGGAAAACUGGACUU |
| miR-145-5p mimic control sense | UUCUCCGAACGUGUCACGUTT |
| miR-145-5p mimic control antisense | ACGUGACACGUUCGGAGAATT |
| miR-145-5p inhibitor | AGGGAUUCCUGGGAAAACUGGAC |
| miR-145-5p inhibitor control | CAGUACUUUUGUGUAGUACAA |
| sh-ERK2 | CAAAGTTCGAGTAGCTATCAA |
| si-SENP2 sense | CUCCUAAUGGAAUAAGCGATT |
| si-SENP2 antisense | UCGCUUAUUCCAUUAGGAGTT |

**Table S2. Primers for the construction of plasmids.**

| Plasmid | Sequence (5’-3’) |
| --- | --- |
| ERK2-K99R F | CCATCGAGCAAATGAGAGATGTATATATAGTACAGGACCTCATGGAAACAG |
| ERK2-K99R R | CATCTCTCATTTGCTCGATGGTTGGTGCTCGAATAATGTCA |
| ERK2-K272R F | CACAAAAATAGAGTGCCATGGAACAGGCTGTTCCCAAATGCT |
| ERK2-K272R R | CCATGGCACTCTATTTTTGTGTGGAAGAGAAAGCAAATAGTTCCTAGC |
| ERK2-K330R F | agcAccattcAGAttcgacatggaattggatgacttgcctAAG |
| ERK2-K330R R | ATGTCGAATCTGAATGGTGCTTCGGCGATGGGCTCGTCA |
| pCDH-ERK2 F | TTAGCTAGCATGGCGGCGGCGGGC |
| pCDH-ERK2 R | ATTTGCGGCCGCTTAAGATCTGTATCCT |
| pCDH-ERK2 K99R&K272R F | TTAGCTAGCATGGCGGCGGCGGGC |
| pCDH-ERK2 K99R&K272R R | ATTTGCGGCCGCTTAAGATCTGTATCCT |
| pCDH-ERK2w/m^R^ F | AACAAGGTAAGAGTCGCAATAAAGAAAATCAGCCCCTTTGAGCAC |
| pCDH-ERK2w/m^R^ R | GATTGCAACGCGTACCTTGTTGACATTATCATAAGCAGAGCAACA |

**Table S3. Recombinant DNA.**

| Plasmid | Source | Identifier |
| --- | --- | --- |
| 3XFLAG | Sigma-Aldrich | E4026 |
| FLAG-ERK2 | Provided by Dr. Qiao Wu in Xiamen University | N/A |
| FLAG-ERK2 K99R | This paper | N/A |
| FLAG-ERK2 K272R | This paper | N/A |
| FLAG-ERK2 K330R | This paper | N/A |
| FLAG-ERK2 K99R&K330R | This paper | N/A |
| pCDH-ERK2 | This paper | N/A |
| pCDH-ERK2 K99R&K330R | This paper | N/A |
| pCDH-Puro | [System Biosciences](http://www.integratedsci.com.au/brands/system-biosciences.html) | CD510B-1 |
| pCDH-SENP2 | Provided by Dr. Jinke Cheng in Shanghai Jiao Tong University | N/A |
| pGreen-Puro | [System Biosciences](http://www.integratedsci.com.au/brands/system-biosciences.html) | SI505A-1 |
| pGreen-sh-SENP2 | Provided by Dr. Jinke Cheng in Shanghai Jiao Tong University | N/A |
| pGreen-sh-ERK2 | This paper | N/A |
| RGS-His-SENP2w | This paper | N/A |
| RGS-His-SENP2m | This paper | N/A |
| HA-SUMO1 | Addgene | 17359 |
| HA-SUMO2 | This paper | N/A |

**Table S4. Primers for quantitative real-time PCR.**

| Primer | Sequence (5’-3’) |
| --- | --- |
| 18S F | CCGTTGTGATCCTGCCAGTA |
| 18S R | GCTTGATCCTTCTGCAGGTT |
| SENP2 F | CAGAGACGATGGTCGGAATCAG |
| SENP2 R | CCTCCTGAGTAAGCCATTGCTTC |
| ERK2 F | AAAGACCGGAAGCCATTACAAG |
| ERK2 R | GCCACTGAGAAGTGTCTAATTGA |
| miR-145-5p RT | GTCGTATCCAGTGCAGGGTCCGAGGTATTCGCACTGGATACGACAGGGATT |
| miR-145-5p F | GCGTCCAGTTTTCCCAGG |
| miR-145-5p R | GCAGGGTCCGAGGTATTC |
| U6 RT | CGCTTCACGAATTTGCGTGTCAT |
| U6 F | GCTTCGGCAGCACATATACTAAAAT |
| U6 R | CGCTTCACGAATTTGCGTGTCAT |

**Table S5. Antibodies for Western blotting and co-IP assay.**

| Name | Identifier | Source | Species | Dilution |
| --- | --- | --- | --- | --- |
| β-Actin | AB0011 | Abways | Mouse | 1/5000 |
| β-Tubulin | 10094-1-AP | Proteintech | Mouse | 1/10000 |
| E-cadherin | 20874-1-AP | Proteintech | Rabbit | 1/1000 |
| ERK2 | sc-1647 | Santa Cruz | Mouse | 1/500 |
| ERK1/2 | 4695 | Cell Signaling | Rabbit | 1/1000 |
| fibronectin | 15613-1-AP | Proteintech | Rabbit | 1/1000 |
| FLAG | F1804 | Sigma-Aldrich | Mouse | 1/1000 |
| HA | 3724 | Cell Signaling | Rabbit | 1/1000 |
| Normal mouse IgG-HRP | sc-2748 | Santa Cruz | Mouse | 1/5000 |
| Normal Rabbit IgG-HRP | sc-2750 | Santa Cruz | Rabbit | 1/5000 |
| occludin | 2726017-AP | Proteintech | Rabbit | 1/1000 |
| p-ERK1/2 | CY5277 | Abways | Rabbit | 1/1000 |
| SENP2 | sc-67075 | Santa Cruz | Rabbit | 1/1000 |
| SENP2 | Ab58418 | Abcam | Rabbit | 1/1000 |
| SUMO2 | ab81371 | Abcam | Mouse | 1/1000 |
| N-cadherin | 22018-1-AP | Proteintech | Rabbit | 1/1000 |
| vimentin | 10366-1-AP | Proteintech | Rabbit | 1/1000 |
| ZEB1 | 21544-1-AP | Proteintech | Rabbit | 1/1000 |
| ZEB2 | 14026-1-AP | Proteintech | Rabbit | 1/1000 |

**Table S6. Chemicals and reagents.**

| Chemical | Source | Identifier |
| --- | --- | --- |
| Breast cancer chips | Shanghai Well Biotechnology | ZL-Brc3N961 |
| Ampicillin | Sigma-Aldrich | A9518 |
| Cycloheximide | Sigma-Aldrich | 239763-M |
| DAPI | Sigma-Aldrich | D9542 |
| FD Cresyl Violet solution | FD Neuro Technologies | PS102-01 |
| Goat serum | Yeasen | 36119ES03 |
| Kanamycin | Sigma-Aldrich | BP861 |
| Lipofectamine 3000 | Invitrogen | L3000075 |
| Matrigel | Corning | 354234 |
| MG132 | Selleck | S2619 |
| MTT | Sigma-Aldrich | M2128-100MG |
| N-Ethylmaleimide | Sigma-Aldrich | E3876 |
| PMSF | Sigma-Aldrich | 10837091001 |
| Paraformaldehyde | Sigma-Aldrich | 158127 |
| Prolong gold antifade mountant | Invitrogen | P36930 |
| Protease inhibitor cocktail | Targetmol | C0001 |
| Protein A/G agarose Beads | Yeasen | 36403ES25 |
| Puromycin Dihydrochloride | Ameresco | J593-25MG |
| Q5 DNA polymerase | New England Biolabs | M0492S |
| T4 DNA ligase | New England Biolabs | M0202S |
| Transwell plate | Corning | CLS3422-48EA |

**Table S7. Information of ten breast cancer patients.**

| **Type** | **Pathology number** | **Age** | **TNM** | **ER** | **PR** | **HER2** | **Ki-67 (%)** |
| --- | --- | --- | --- | --- | --- | --- | --- |
| Cancer | 2021-17389-3 | 67 | T1N0Mx | - | + | + | 60 |
| Paracancerous | 2021-17389-5 | 67 |  |  |  |  |  |
| Cancer | 2021-17315-6 | 50 | T2 | + | + | + | 30 |
| Paracancerous | 2021-17315-9 | 50 |  |  |  |  |  |
| Cancer | 2021-16544-6 | 46 | T2 | + | + | + | 60 |
| Paracancerous | 2021-16544-11 | 46 |  |  |  |  |  |
| Cancer | 2021-15272-5 | 62 | T2 | + | + | + | 50 |
| Paracancerous | 2021-15272-9 | 62 |  |  |  |  |  |
| Cancer | 2021-15253-7 | 65 | T2NxMx | + | - | - | 30 |
| Paracancerous | 2021-15253-12 | 65 |  |  |  |  |  |
| Cancer | 2021-14673-6 | 50 | T1c | + | + | + | 20 |
| Paracancerous | 2021-14673-11 | 50 |  |  |  |  |  |
| Cancer | 2021-14548-3 | 49 | T1c | + | + | + | 80 |
| Paracancerous | 2021-14548-6 | 49 |  |  |  |  |  |
| Cancer | 2021-11773-8 | 57 | T1cNxMx | + | + | + | 20 |
| Paracancerous | 2021-11773-11 | 57 |  |  |  |  |  |
| Cancer | 2021-07758-4 | 55 | T1c | + | + | + | 1 |
| Paracancerous | 2021-07758-6 | 55 |  |  |  |  |  |
| Cancer | 2021-03089-11 | 49 | T2 | + | + | + | 30 |
| Paracancerous | 2021-03089-12 | 49 |  |  |  |  |  |

**Table S8. Information of breast cancer chip.**

| **Position** | **Type** | **Pathology number** | **Age** | **TNM** | **Stage** | **ER** | **PR** | **HER2** | **Ki-67 (%)** |
| --- | --- | --- | --- | --- | --- | --- | --- | --- | --- |
| A01 | Cancer | 15-00787 | 53 | T2N0M0 | Ⅱ | - | - | - | 70 |
| A02 | Paracancerous | 15-00787 |  |  |  |  |  |  |  |
| A03 | Cancer | 15-00982 | 50 | T1cN0M0 | Ⅰ | - | - | - | 70 |
| A04 | Paracancerous | 15-00982 |  |  |  |  |  |  |  |
| A05 | Cancer | 15-01107 | 51 | T1cN0M0 | Ⅰ | - | - | - | 60 |
| A06 | Paracancerous | 15-01107 |  |  |  |  |  |  |  |
| A07 | Cancer | 15-05400 | 59 | T1cN0M0 | Ⅰ | - | - | + | 50 |
| A08 | Paracancerous | 15-05400 |  |  |  |  |  |  |  |
| A09 | Cancer | 15-06761 | 75 | T2N0M0 | Ⅱ | - | - | + | 80 |
| A10 | Paracancerous | 15-06761 |  |  |  |  |  |  |  |
| A11 | Cancer | 15-07038 | 57 | T2N0M0 | Ⅱ | - | - | - | 80 |
| A12 | Paracancerous | 15-07038 |  |  |  |  |  |  |  |
| B01 | Cancer | 15-11818 | 47 | T2N2M0 | Ⅲ | - | - | + | 40 |
| B02 | Paracancerous | 15-11818 |  |  |  |  |  |  |  |
| B03 | Cancer | 15-12662 | 91 | T2NxM0 | Ⅲ | - | - | - | 50 |
| B04 | Paracancerous | 15-12662 |  |  |  |  |  |  |  |
| B05 | Cancer | 15-12665 | 69 | T2N2M0 | Ⅲ | - | - | + | 50 |
| B06 | Paracancerous | 15-12665 |  |  |  |  |  |  |  |
| B07 | Cancer | 15-13946 | 51 | T1cN0M0 | Ⅰ | - | - | + | 40 |
| B08 | Paracancerous | 15-13946 |  |  |  |  |  |  |  |
| B09 | Cancer | 15-16154 | 34 | T3N3M0 | Ⅲ | - | - | - | 70 |
| B10 | Paracancerous | 15-16154 |  |  |  |  |  |  |  |
| B11 | Cancer | 15-15482 | 62 | T1cN0M0 | Ⅰ | - | - | + | 20 |
| B12 | Paracancerous | 15-15482 |  |  |  |  |  |  |  |
| C01 | Cancer | 15-20581 | 74 | T2N0M0 | Ⅱ | - | - | - | 40+ |
| C02 | Paracancerous | 15-20581 |  |  |  |  |  |  |  |
| C03 | Cancer | 15-28308 | 65 | T2N0M0 | Ⅱ | - | - | - | 30 |
| C04 | Paracancerous | 15-28308 |  |  |  |  |  |  |  |
| C05 | Cancer | 15-32226 | 45 | T2N0M0 | Ⅱ | - | - | - | 80 |
| C06 | Paracancerous | 15-32226 |  |  |  |  |  |  |  |
| C07 | Cancer | 16-04985 | 60 | T2N0M0 | Ⅱ | - | - | - | 70 |
| C08 | Paracancerous | 16-04985 |  |  |  |  |  |  |  |
| C09 | Cancer | 16-07115 | 58 | T2N1M0 | Ⅱ | - | - | - | 60 |
| C10 | Paracancerous | 16-07115 |  |  |  |  |  |  |  |
| C11 | Cancer | 16-06423 | 56 | T2N0M0 | Ⅱ | - | - | - | 70 |
| C12 | Paracancerous | 16-06423 |  |  |  |  |  |  |  |
| D01 | Cancer | 16-08457 | 85 | T2NxM0 | Ⅲ | - | - | - | 60 |
| D02 | Paracancerous | 16-08457 |  |  |  |  |  |  |  |
| D03 | Cancer | 16-12704 | 68 | T2N0M0 | Ⅱ | - | - | - | 40 |
| D04 | Paracancerous | 16-12704 |  |  |  |  |  |  |  |
| D05 | Cancer | 16-15296 | 85 | T2NxM0 | Ⅲ | - | - | + | 50 |
| D06 | Paracancerous | 16-15296 |  |  |  |  |  |  |  |
| D07 | Cancer | 16-19339 | 43 | T2N0M0 | Ⅱ | - | - | - | 60 |
| D08 | Paracancerous | 16-19339 |  |  |  |  |  |  |  |
| D09 | Cancer | 16-25345 | 48 | T1cN1M0 | Ⅱ | - | - | + | 80 |
| D10 | Paracancerous | 16-25345 |  |  |  |  |  |  |  |
| D11 | Cancer | 16-32642 | 33 | T2N1M0 | Ⅱ | - | - | - | 80 |
| D12 | Paracancerous | 16-32642 |  |  |  |  |  |  |  |
| E01 | Cancer | 17-01841 | 49 | T1cN0M0 | Ⅰ | - | - | + | 60 |
| E02 | Paracancerous | 17-01841 |  |  |  |  |  |  |  |
| E03 | Cancer | 17-13034 | 84 | T2NxM0 | Ⅲ | - | - | + | 15 |
| E04 | Paracancerous | 17-13034 |  |  |  |  |  |  |  |
| E05 | Cancer | 17-13586 | 57 | T2N3M0 | Ⅲ | - | - | - | 20 |
| E06 | Paracancerous | 17-13586 |  |  |  |  |  |  |  |
| E07 | Cancer | 17-14367 | 32 | T1cN0M0 | Ⅰ | - | - | + | 45 |
| E08 | Paracancerous | 17-14367 |  |  |  |  |  |  |  |
| E09 | Cancer | 17-15464 | 61 | T1cN1M0 | Ⅱ | - | - | - | 85 |
| E10 | Paracancerous | 17-15464 |  |  |  |  |  |  |  |
| E11 | Cancer | 17-19496 | 36 | T1cNxM0 | Ⅰ | - | - | - | 60 |
| E12 | Paracancerous | 17-19496 |  |  |  |  |  |  |  |
| F01 | Cancer | 17-20170 | 55 | T2N1M0 | Ⅱ | - | - | + | 75 |
| F02 | Paracancerous | 17-20170 |  |  |  |  |  |  |  |
| F03 | Cancer | 17-20559 | 49 | T1cN0M0 | Ⅰ | - | - | + | 45 |
| F04 | Paracancerous | 17-20559 |  |  |  |  |  |  |  |
| F05 | Cancer | 17-24831 | 70 | T2N1M0 | Ⅱ | - | - | - | 7 |
| F06 | Paracancerous | 17-24831 |  |  |  |  |  |  |  |
| F07 | Cancer | 17-25081 | 33 | T2N0M0 | Ⅱ | - | - | Weak + | 80 |
| F08 | Paracancerous | 17-25081 |  |  |  |  |  |  |  |
| F09 | Cancer | 17-27624 | 56 | T1cN0M0 | Ⅰ | - | - | + | 70 |
| F10 | Paracancerous | 17-27624 |  |  |  |  |  |  |  |
| F11 | Cancer | 17-27617 | 66 | T2N2M0 | Ⅲ | - | - | - | 90 |
| F12 | Paracancerous | 17-27617 |  |  |  |  |  |  |  |
| G01 | Cancer | 17-27723 | 77 | T1cN0M0 | Ⅰ | - | - | - | 60 |
| G02 | Paracancerous | 17-27723 |  |  |  |  |  |  |  |
| G03 | Cancer | 17-30636 | 61 | T1cN0M0 | Ⅰ | - | - | + | 30 |
| G04 | Paracancerous | 17-30636 |  |  |  |  |  |  |  |
| G05 | Cancer | 18-00631 | 60 | T3N0M0 | Ⅲ | - | - | + | 60 |
| G06 | Paracancerous | 18-00631 |  |  |  |  |  |  |  |
| G07 | Cancer | 17-31005 | 63 | T2N0M0 | Ⅱ | - | - | - | 30 |
| G08 | Paracancerous | 17-31005 |  |  |  |  |  |  |  |
| G09 | Cancer | 17-35186 | 32 | T1cN3M0 | Ⅲ | - | - | - | 30 |
| G10 | Paracancerous | 17-35186 |  |  |  |  |  |  |  |
| G11 | Cancer | 18-00280 | 58 | T1cN0M0 | Ⅰ | - | - | + | 20 |
| G12 | Paracancerous | 18-00280 |  |  |  |  |  |  |  |
| H01 | Cancer | 18-10407 | 68 | T1cN2M0 | Ⅲ | - | - | + | 30 |
| H02 | Paracancerous | 18-10407 |  |  |  |  |  |  |  |
| H03 | Cancer | 18-12275 | 41 | T2N0M0 | Ⅱ | - | - | + | 60 |
| H04 | Paracancerous | 18-12275 |  |  |  |  |  |  |  |
| H05 | Cancer | 18-11577 | 33 | T3N0M0 | Ⅲ | - | - | + | 90 |
| H06 | Paracancerous | 18-11577 |  |  |  |  |  |  |  |
| H07 | Cancer | 18-12550 | 61 | T2N0M0 | Ⅱ | - | - | - | 55 |
| H08 | Paracancerous | 18-12550 |  |  |  |  |  |  |  |
| H09 | Cancer | 18-13581 | 47 | T1bN0M0 | Ⅰ | - | - | - | 50 |
| H10 | Paracancerous | 18-13581 |  |  |  |  |  |  |  |
| H11 | Cancer | 18-13897 | 61 | T1cN0M0 | Ⅰ | - | - | - | 75 |
| H12 | Paracancerous | 18-13897 |  |  |  |  |  |  |  |

**Supplemental figure legends**

**Figure S1. SENP2 was highly correlated with the progression of breast cancer patients.**

SENP2 in breast cancer and precancerous tissue chips from breast cancer patients was analyzed by immunohistochemistry with the anti-SENP2 antibody (n = 48 biological repeats/ group). The scale bar is 200 μm.

**Figure S2. SENP2 knockdown inhibited the EMT process in breast cancer cells.**

A-B. SENP2 was stably knocked down in MCF-7 and MDA-MB-231 cells. Two sh-SENP2 or control lentiviruses were transfected into two breast cancer cells, and the cell lysates of stably constructed cells were detected by real-time PCR (A) or IB with anti-SENP2 and anti-β-Tubulin antibodies (B, left). The results of the quantitative analysis of Western blotting are shown in the right panel (B, right, n = 3 biological repeats/group).

C. Expression levels of EMT gene transcripts were detected after stable SENP2 knockdown in MDA-MB-231 cells. The expression levels of EMT gene transcripts in MDA-MB-231 cells were measured by real-time PCR and normalized to control cells (n = 3 biological and 3 technical repeats/group).

D. SENP2 elimination showed no significant effect on body weight. The body weight was monitored every four days until the end of the experiment (n = 5 mice/group).

**Figure S3. SENP2 over-expression promoted the progression of breast cancer *in vitro*.**

A-B. SENP2 was stably overexpressed in MCF-7 and MDA-MB-231 cells. pCDH-SENP2 or control lentivirus was transfected into two breast cancer cells, and the cell lysates of stably constructed cells were detected by real-time PCR (A) or IB with anti-SENP2 and anti-β-Tubulin antibodies (B, left). The results of the quantitative analysis of Western blotting are shown in the right panel (B, n = 3 biological repeats/group).

C. SENP2 overexpression promoted the proliferation of MCF7 (left) and MDA-MB-231 (right) cells. Growth curves of SENP2 knockdown or control cells were constructed from daily quantification of cell numbers (n = 3 biological repeats/group).

D. SENP2 overexpression promoted the colony formation of MCF7 and MDA-MB-231 cells. Cells were seeded in 24-well plates for 14 days and stained with crystal violet for the colony formation assay (left). The colony formation ability was analyzed (n = 3 biological repeats/group).

E. SENP2 overexpression promoted the migration of MCF7 and MDA-MB-231 cells. Cell migration was determined by wound-healing assay in stably transfected cells (left). The migration rate was measured and analyzed (right, n = 3 biological repeats/group). The scale bar is 200 μm.

F. SENP2 overexpression promoted the invasion of MCF7 and MDA-MB-231 cells. Cell invasion was analyzed by transwell assay in stably transfected cells (left). The invasive cells were measured and analyzed (right, n = 3 biological repeats/group). The scale bar is 200 μm.

G. SENP2 overexpression promoted EMT in MCF7 and MDA-MB-231 cells. pCDH-Ctrl and pCDH-SENP2 plasmids were packaged and transfected into cells, and the cell lysates were detected by IB with the indicated antibodies.

**Figure S4. The knockdown of ERK2 inhibits the progression of breast cancer *in vitro*.**

A. The levels of ERK1 showed no significant difference between various subtypes of breast cancer tissues. ERK1 expression in different subtypes of breast cancers was analyzed in the GEPIA database.

B. The relationship between ERK2 expression and survival time in patients with various breast cancer subtypes, including TNBC, Luminal A, Luminal B, and HER2+, were analyzed by survival analysis with the Kaplan-Meier Plotter database.

C. ERK2 was stably knocked down in MDA-MB-231 and MCF-7 cells. sh-ERK2 or control lentivirus was transfected into breast cancer cells, and the cell lysates of stably constructed cells were detected by IB with anti-ERK2 and anti-β-Tubulin antibodies (left). The results of the quantitative analysis of Western blotting are shown in the right panel (right, n = 3 biological repeats/group).

D. ERK2 knockdown inhibited the growth of MDA-MB-231 and MCF-7 cells. sh-ERK2 or control lentivirus was transfected into breast cancer cells, and the growth curves of ERK2 knockdown or control cells were constructed from daily quantification of cell numbers (n = 3 biological repeats/group).

E. ERK2 knockdown inhibited the colony formation of MDA-MB-231 and MCF-7 cells. Cells were seeded in 24-well plates for 14 days and stained with crystal violet for the colony formation assay (left). The colony formation ability was analyzed (right, n = 3 biological repeats/group).

F. ERK2 knockdown inhibited the migration of MDA-MB-231 and MCF-7 cells. Cell migration was determined by wound-healing assay in stably transfected cells (left). The migration rate was measured and analyzed (right, n = 3 biological repeats/group). The scale bar is 200 μm.

G. ERK2 knockdown inhibited the invasion of MDA-MB-231 and MCF-7 cells. Cell migration and invasion were analyzed by transwell assay in stably transfected cells (left). The migration and invasion of cells were measured and analyzed (right, n = 5 biological repeats/group). The scale bar is 200 μm.

H. ERK2 knockdown inhibited EMT in MDA-MB-231 and MCF-7 cells. The sh-NC and sh-ERK2 plasmids were packaged and transfected into cells, and the cell lysates were detected by IB with the indicated antibodies.

I. ERK2 overexpression rescued the proliferation inhibited by SENP2 knockdown in MDA-MB-231 cells. The growth curves of stably transfected cells were constructed from the daily quantification of cell numbers (n = 3 biological repeats/group).

J. ERK2 overexpression rescued the colony formation inhibited by SENP2 knockdown in MDA-MB-231 cells. Cells were seeded in 24-well plates for 14 days and stained with crystal violet for the colony formation assay (left). The colony formation ability was analyzed (n = 3 biological repeats/group).

K. ERK2 overexpression rescued the migration inhibited by SENP2 knockdown in MDA-MB-231 cells. Cell migration was determined by wound-healing assay in stably transfected cells (left). The migration rate was measured and analyzed (right, n = 3 biological repeats/group). The scale bar is 200 μm.

L. ERK2 overexpression promoted the EMT process inhibited by SENP2 knockdown in MDA-MB-231 cells. The whole cell lysates were detected by IB with the indicated antibodies.

**Figure S5. The predicted SUMOylation sites of ERK2.**

A. The protein expression level of ERK2 was decreased in SENP2^−/−^ MEF cells. The cell lysates of SENP2^+/+^ and SENP2^−/−^ MEF cells were analyzed by IB with the indicated antibodies (left), and the results of the quantitative analysis of Western blotting were shown in the right panel (n = 3 biological repeats/group).

B. SENP2 positively regulated the expression of ERK2 in MCF-7 cells. Two sh-SENP2 or control lentiviruses were transfected into MCF-7 cells, and the cell lysates of stably constructed cells were detected by IB with the indicated antibodies (left). The results of the quantitative analysis of Western blotting were shown in the right panel (n = 3 biological repeats/group).

C. The mRNA level of ERK2 was not significant different between SENP2^+/+^ and SENP2^-/-^ MEF cells. The expression levels of SENP2 and ERK2 transcripts in SENP2^-/-^ MEF cells were measured by real-time PCR and normalized to the control (n = 3 biological repeats/group).

D. The SUMOylation sites of ERK2 were predicted by the SUMOplot database.

E. The predicted SUMOylation sites K99 and K330 were highly conserved in various species.

**Figure S6. SUMO site mutant ERK2 enhanced the increased cell growth and metastasis induced by wild-type ERK2.**

A. SUMO mutant ERK2 enhanced the proliferation of MDA-MB-231 cells. Growth curves of wild-type or SUMO mutant ERK2 cells were generated from daily quantification of cell numbers (n = 3 biological repeats/group).

B. SUMO mutant ERK2 enhanced the colony formation of MDA-MB-231 cells. Cells were seeded for 14 days and stained with 0.1% crystal violet for the colony formation assay (left). The colony formation ability was analyzed (n = 3 biological repeats/group).

C. SUMO mutant ERK2 enhanced the migration of MDA-MB-231 cells. Cell migration was detected by wound healing assay in stably transfected cells (left). The migration rate was measured and analyzed (right, n = 3 biological repeats/group). The scale bar is 200 μm.

D. SUMO mutant ERK2 enhanced the invasion of MDA-MB-231 cells. Cellular invasiveness was assessed via the transwell assay in cells stably transfected with the construct (left). The invasive cells were measured and analyzed (right, n = 4 repeats/group). The scale bar represents 200 μm.

E. SUMOylation of ERK2 regulates the EMT process in breast cancer cells. Wild-type or SUMOylation site mutant ERK2 was stably expressed in MDA-MB-231 cells, and whole cell lysates were detected by Western blotting with the indicated antibodies.

**Figure S7. miR-145-5p was predicted to interact with SENP2.**

A. miR-145-5p interacted with the 3'UTR of SENP2.The predicted potential interaction sites between the 3'UTR of SENP2 mRNA and miR-145-5p were obtained from the miRBase and TargetScan databases.

B. The binding sites of the 3'UTR of SENP2 were mutated.
